# An Improved Moth-Flame Optimization Algorithm with Adaptation Mechanism to Solve Numerical and Mechanical Engineering Problems

**DOI:** 10.3390/e23121637

**Published:** 2021-12-06

**Authors:** Mohammad H. Nadimi-Shahraki, Ali Fatahi, Hoda Zamani, Seyedali Mirjalili, Laith Abualigah

**Affiliations:** 1 Faculty of Computer Engineering, Najafabad Branch, Islamic Azad University, Najafabad 8514143131, Iran; fatahi.ali.edu@sco.iaun.ac.ir (A.F.); hoda_zamani@sco.iaun.ac.ir (H.Z.); 2 Big Data Research Center, Najafabad Branch, Islamic Azad University, Najafabad 8514143131, Iran; 3 Centre for Artificial Intelligence Research and Optimisation, Torrens University Australia, Brisbane 4006, Australia; 4 Yonsei Frontier Lab, Yonsei University, Seoul 03722, Korea; 5 Faculty of Computer Sciences and Informatics, Amman Arab University, Amman 11953, Jordan; laythdyabat@aau.edu.jo; 6 School of Computer Sciences, Universiti Sains Malaysia, Pulau Pinang 11800, Malaysia

**Keywords:** optimization, metaheuristic algorithms, swarm intelligence algorithm, moth-flame optimization, mechanical engineering problems

## Abstract

Moth-flame optimization (MFO) algorithm inspired by the transverse orientation of moths toward the light source is an effective approach to solve global optimization problems. However, the MFO algorithm suffers from issues such as premature convergence, low population diversity, local optima entrapment, and imbalance between exploration and exploitation. In this study, therefore, an improved moth-flame optimization (I-MFO) algorithm is proposed to cope with canonical MFO’s issues by locating trapped moths in local optimum via defining memory for each moth. The trapped moths tend to escape from the local optima by taking advantage of the adapted wandering around search (AWAS) strategy. The efficiency of the proposed I-MFO is evaluated by CEC 2018 benchmark functions and compared against other well-known metaheuristic algorithms. Moreover, the obtained results are statistically analyzed by the Friedman test on 30, 50, and 100 dimensions. Finally, the ability of the I-MFO algorithm to find the best optimal solutions for mechanical engineering problems is evaluated with three problems from the latest test-suite CEC 2020. The experimental and statistical results demonstrate that the proposed I-MFO is significantly superior to the contender algorithms and it successfully upgrades the shortcomings of the canonical MFO.

## 1. Introduction

In the majority of real-world optimization problems, a large number of decision variables are interacted with together, which is a very time-consuming process for finding an exact solution [1,2,3,4,5,6,7]. Metaheuristic algorithms have been widely used in recent years to approximate near-optimal solutions for real-world problems in various applications such as discrete optimization [8,9,10,11,12,13,14,15,16,17], continuous optimization [18,19,20,21,22], and constrained engineering problems [23,24,25,26,27,28,29,30,31,32,33]. Moreover, a novel research field has emerged in this area which successfully combines machine learning and swarm intelligence approaches to obtain outstanding results in different areas [34,35,36]. Metaheuristic algorithms can be classified into two main categories of non-nature-inspired and nature-inspired [2]. Simulated annealing (SA) [37], tabu search (TS) [38], adaptive dimensional search (ADS) [39], and iterated local search (ILS) [40] are some well-known non-nature-inspired metaheuristic algorithms. Although these algorithms have demonstrated remarkable local search capabilities, they may easily be trapped in local optimum in complex problems [2,3].

Nature-inspired algorithms consist of three main categories: evolutionary, physics-based, and swarm intelligence (SI). Evolutionary algorithms are mostly inspired by Darwin’s theory of evolution. Some examples are: genetic algorithm (GA) [41,42], genetic programming (GP) [43], differential evolution (DE) [44], evolution strategy (ES) [45], and quantum-based avian navigation optimizer (QANA) [46]. Ensemble of mutation strategies and parameters in differential evolution (EPSDE) algorithm [47], multi-population ensemble DE (MPEDE) [48], ensemble of differential evolution variants (EDEV) [49], and multi-trial vector-based differential evolution (MTDE) [50] are some successful improvements on evolutionary algorithms. Physics-based algorithms propose meaningful search strategies inspired by physics and mathematics laws to solve optimization problems. The big bang–big crunch (BB-BC) [51], charged system search (CSS) [52], ray optimization (RO) [53], atom search optimization (ASO) [54], arithmetic optimization algorithm (AOA) [55], and atomic orbital search (AOS) [56] are some of the successful physics-based algorithms in the literature.

Swarm intelligence (SI) algorithms are mainly derived from the social interaction behavior of terrestrial animals, aquatic animals, birds, and insects in nature [57]. Grey wolf optimizer (GWO) [58], chimp optimization algorithm (ChOA) [59], and gorilla troops optimizer (GTO) [60] are inspired by the behavior of terrestrial animals to solve optimization problems. Despite their simplicity and broad use, they may suffer from common drawbacks such as low population diversity, sinking into local optimum, and premature convergence problems. Therefore, there have been many improvements with different approaches applied to overcome their weaknesses [61,62,63,64]. Some SI algorithms are inspired by the behavior of aquatic animals such as prey besieging and foraging, which have been modeled in krill herd (KH) [65], dolphin echolocation (DE) [66], and whale optimization algorithm (WOA) [67]. The social intelligence behaviors of birds and insects encourage researchers to propose a new generation of SI algorithms that are modeled by foraging, hunting, and navigation behavior. Ant colony optimization (ACO) [68], ant lion optimizer (ALO) [69], social spider algorithm (SSA) [70], crow search algorithm (CSA) [71], African vultures optimization algorithm (AVOA) [72], Aquila optimizer (AO) [73], and moth-flame optimization (MFO) [74] are popular SI algorithms inspired by the behaviors of birds and insects.

The moth-flame optimizer (MFO) is a prominent SI algorithm inspired by the moths’ locomotion toward the light source. The MFO is appealing because of its ease of implementation, acceptable time complexity, and small number of parameters, which make it applicable in real-world optimization problems such as image segmentation [75,76,77], feature selection [78,79,80,81,82], food processing [83,84,85], and engineering optimization [86,87,88,89,90,91]. Consequently, many MFO variants have been developed such as Lévy-flight moth-flame optimization (LMFO) [92], non-dominated sorting moth flame optimization (NS-MFO) [93], enhanced moth-flame optimization (EMFO) [94], water cycle–moth-flame optimization (WCMFO) [95], and sine-cosine moth-flame optimization (SMFO) [96]. However, MFO and its variants cannot satisfy the needs of the optimization process for challenging problems, and they still suffer from some weaknesses such as low population diversity [97,98], premature convergence, local optima trapping [99,100], and imbalance between exploration and exploitation [101]. The main reason for these MFO’s weaknesses is that the majority of moths are trapped in the local optima in the early iterations which results in low population diversity. The question of this study is how moths can escape the local optima trapping and be moved to promising zones?

This paper proposes an improved MFO algorithm named I-MFO which uses moths’ memory mechanism and an adapted version of the wandering around search (WAS) strategy introduced in our prior study [57] to find and possibly free trapped moths. In the I-MFO algorithm, trapped moths are detected by comparing their best experienced flame fitness (*Fbest*) with their current positions’ fitness (*OM*). If the current position of each moth is not better than its memory, the moth is considered to be a trapped moth, and the adapted wandering around search (AWAS) strategy is employed to possibly free it from local optima by performing some random short flights, which also leads to amelioration of the premature convergence. Moreover, the *fl* parameter is used to strike a balance between exploration and exploitation by limiting the moths’ flight range. In the end, the new position and its fitness value replace the previous ones if the new fitness value is better than its *Fbest*.

The proposed I-MFO is comprehensively investigated by the benchmark functions CEC 2018 [102] in different dimensions of 30, 50, and 100, and compared with the state-of-the-art metaheuristic algorithms and three MFO variants including SA, continuous genetic algorithm (CGA) [42], GWO, MFO, WOA, LMFO, WCMFO, ChOA, AOA, and SMFO. The results demonstrate that the I-MFO can avoid local optima trapping, maintain population diversity, mitigate premature convergence, and strike a balance between exploration and exploitation. The I-MFO algorithm is statistically evaluated by the Friedman test and post hoc analysis to prove the superiority of the algorithm. Moreover, the applicability of I-MFO to solve real-world optimization problems was evaluated by three mechanical engineering problems from the latest test-suite CEC 2020 [103]. All experimental evaluations and statistical tests indicate that the I-MFO algorithm outperforms contender algorithms with an overall effectiveness of 92%.

In the rest of this study, the MFO and its variants are discussed in Section 2. Section 3 illustrates the structure of the proposed I-MFO. The results of I-MFO in solving CEC 2018 benchmark test functions are given and analyzed in Section 4, and the results are proven by statistical analysis in Section 5. The applicability of the proposed I-MFO for solving real-world mechanical engineering problems is tested by three problems from the latest test-suite CEC 2020 in Section 6. Finally, conclusions and future directions are given in Section 7.

## 2. Related Works

In this section, the MFO algorithm is first described in detail, and then its variants are reviewed from the perspective of overcoming the MFO weaknesses.

The MFO algorithm proposed in [74] is a population-based SI algorithm that mimics moths’ navigation behavior toward light sources. The moths navigate toward the real-light source (moon) with a straight path and a fixed angle which is called transverse orientation. Moreover, moths are highly attracted to artificial lights such as flames, and because of the close distance, they change their flight angles continuously, which forms a spiral path. This behavior is modeled by the MFO algorithm to solve optimization problems and is reviewed in [104,105,106]. In this algorithm, both moths and flames are considered as solutions. First, moths scatter in the search space randomly and their positions are saved in a matrix *M*, where rows indicate the number of moths (*N*), and columns represent dimensions (*D*). Then, the fitness value of *M* is evaluated and stored in an array *OM* as represented below.

$$M=\left[\begin{array}{cccc} m_{1,1} & m_{1,2} & \cdots & m_{1,D}\\ m_{2,1} & m_{2,2} & \cdots & m_{2,D}\\ \vdots & \vdots & \vdots & \vdots \\ m_{N,1} & m_{N,2} & \cdots & m_{N,D} \end{array}\right]\;\;\;\;\;\;\;\;OM=\left[\begin{array}{c} OM_{1}\\ OM_{2}\\ \vdots \\ OM_{N} \end{array}\right]$$

The positions and fitness values of flames are considered in a matrix *F* and an array *OF*, respectively. In the first iteration, the *OF* is initiated based on ascending order of *OM*, and the corresponding sorted positions are assigned to the matrix *F*. In the next iterations, the *F* will be updated by the best *N* search agents from *F* and current *M* populations. In the optimization process, each moth *M_i_*(*t*) in the current iteration *t* moves around its corresponding flame *F_j_* using a logarithmic spiral defined in Equation (1), where *Dis_i_* is computed by Equation (2), *b* determines the shape of the logarithmic spiral, and *k* is a random number value between interval (−1, 1).

(1) $$M_{i}\left(t\right)=Dis_{i}.e^{bk}.Cos\left(2\pi k\right)+F_{j}\left(t\right)$$

(2) $$Dis_{i}\left(t\right)=\left|F_{j}\left(t\right)-M_{i}\left(t\right)\right|$$

In this algorithm, the number of flames is computed by Equation (3), where *t* indicates the current iteration, and *N* and *MaxIt* are the number of search agents and the maximum number of iterations, respectively.

(3) $$flame_{no}=round\left(N-t\times \frac{N-1}{MaxIt\;}\right)$$

**Figure 1:**
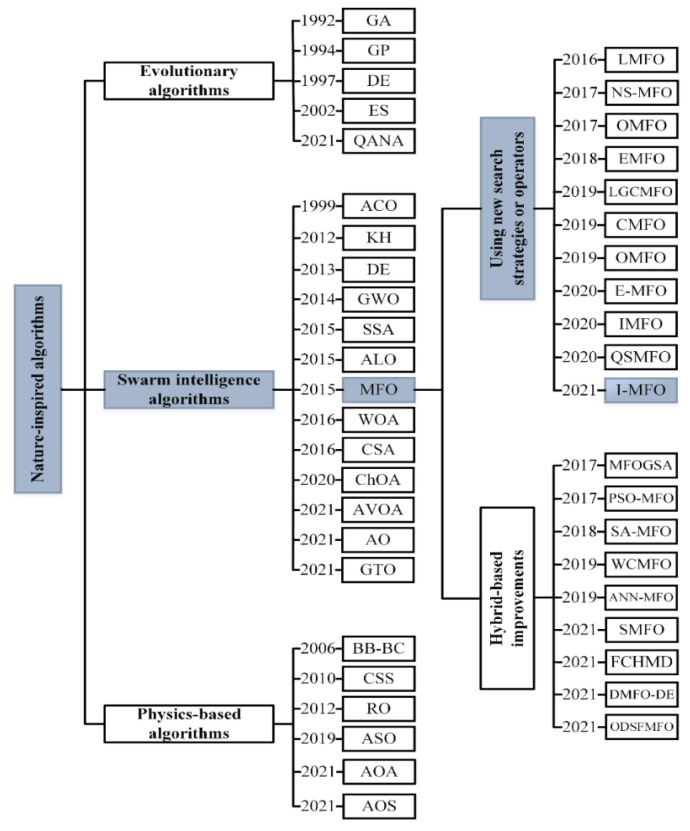
The classification of nature-inspired algorithms and MFO variants.

The MFO algorithm is an effective problem solver that is widely applied for real-world optimization problems. However, MFO is prone to being trapped in local optimum and suffers premature convergence due to its loss of population diversity and imbalance between the two tendencies of exploration and exploitation. Therefore, many variants have been proposed to boost the MFO algorithm, which can be categorized in improved algorithms based on using new search strategies or operators and hybrid-based improvements with other algorithms. Figure 1 shows the classification of SI algorithms and MFO variants.

Many algorithms have been proposed by using new search strategies or operators in the canonical MFO. Li et al. [92] proposed the LMFO algorithm by employing the Lévy-flight strategy to increase the population diversity. Savsani et al. [93] proposed the effective non-dominated moth-flame optimization algorithm (NS-MFO) to solve multi-objective problems using the elitist non-dominated sorting method. The opposition-based moth-flame optimization (OMFO) [107] presents an opposition-based scheme in the canonical MFO to avoid local optimum and increase global exploration. In the EMFO [94], the Gaussian mutation (GM) was added to MFO to increase the diversity. In LGCMFO [100], Xu et al. used new operators such as Gaussian mutation (GM), Lévy mutation (LM), and Cauchy mutation (CM) to boost exploration and exploitation capabilities and encounter the local optima trapping of the MFO. In addition, Hongwei et al. [99] presented the chaos-enhanced moth-flame optimization (CMFO) with ten chaotic maps to cope with the MFO deficiency. Sapre et al. [108] brought up OMFO to cope with premature convergence and local optima trapping by proposing a combination of opposition-based, Cauchy mutation and evolutionary boundary constraint handling. In 2020, Kaur et al. [97] proposed E-MFO by adding a Cauchy mutation (CM) to improve the distribution of the algorithm in the search space. An improved moth-flame optimization (IMFO) [98] algorithm proposes a new flame generation strategy and divides optimization iterations into three phases to encounter low population diversity and enhance MFO’s search balance, respectively. An improved MFO algorithm called QSMFO was proposed by [109] to boost MFO’s exploitation capabilities while enhancing the exploration rate by introducing the simulated annealing strategy and quantum rotation gate, respectively.

Some variants proposed hybrid-based improvements to the MFO algorithm to boost its performance. MFOGSA [83] is a combination of MFO with gravitational search algorithm (GSA) to utilize MFO’s exploration and GSA’s exploitation capabilities. Bhesdadiya et al. [110] proposed a hybrid PSO-MFO algorithm to solve optimization problems. SA-MFO [111] combines MFO with simulated annealing (SA) to overcome local optima trapping and low convergence rate. Khalilpourazari et al. [95] proposed WCMFO to encounter MFO’s entrapping at local optima and low convergence rate, while taking advantage of the water cycle algorithm (WCA). A combination of MFO and artificial neural network (ANN-MFO) was proposed by Singh et al. [112] to solve multi-objective problems in magnetic abrasive finishing of aluminum. Chen et al. [96] introduced SMFO to improve the exploration capability of MFO by integrating it with the sine cosine strategy. An enhanced MFO algorithm was proposed by MP Dang et al. [113] which is a hybridization of MFO and three different methods to solve the design problem of a flexure hinge. Mittal [114] brought up an enhanced moth-flame optimization by integrating MFO and variable neighborhood search to boost search capabilities and convergence accuracy of the canonical MFO. In a recent study, Abd Elaziz et al. [115] proposed the FCHMD algorithm which is a hybridization of Harris hawks optimizer and MFO. In this algorithm, fractional-order Gauss and 2xmod1 chaotic maps are used to generate the initial population. Moreover, the FCHMD algorithm ameliorates premature convergence and stagnation in local optima by applying evolutionary population dynamics. Ahmed et al. [116] brought up DMFO-DE which is a discrete hybrid algorithm developed by integrating differential evolution and MFO to encounter the local optima problem and ameliorate the convergence speed and prevent the local optima problem. Li et al. [117] proposed the ODSFMFO algorithm which consists of an improved flame generation mechanism based on opposition-based learning (OBL) and differential evolution (DE) algorithm, and an enhanced local search mechanism based on shuffled frog leaping algorithm (SFLA) and death mechanism. 

Based on the above review on MFO and its proposed variants, the most serious drawbacks of MFO are premature convergence, getting stuck in local optimum, low population diversity, and deficient balance between exploration and exploitation. Therefore, in this study, the improved moth-flame optimization (I-MFO) algorithm is proposed to encounter MFO’s shortcomings by introducing a memory mechanism and an adapted version of the wandering around search (WAS) strategy [57], called AWAS strategy, to the canonical MFO.

## 3. Proposed Algorithm

**Figure 2:**
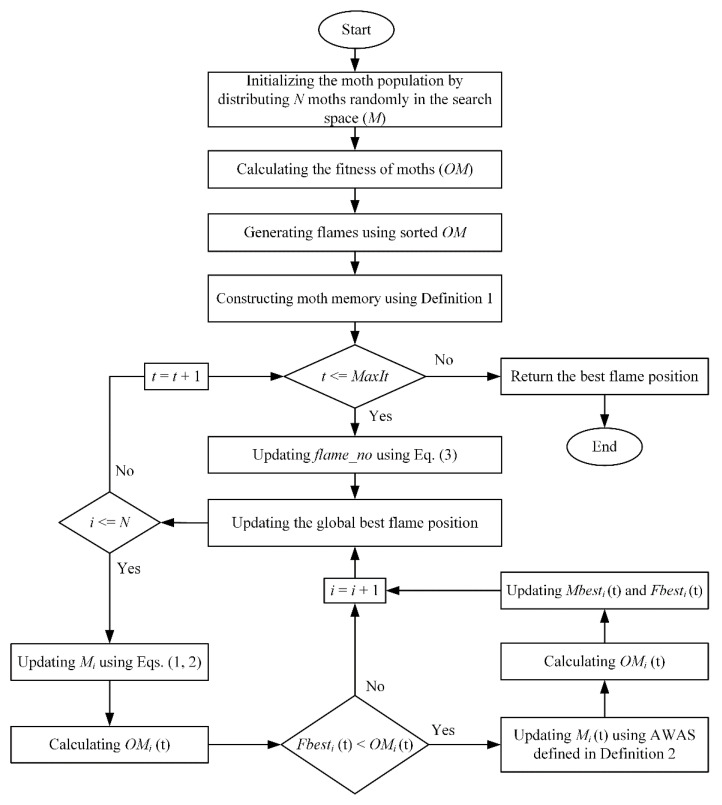
The flowchart of improved moth-flame optimization (I-MFO) algorithm.

The proposed improved moth-flame optimization (I-MFO) algorithm is boosted using a moth memory mechanism and the adapted wandering around search (AWAS) strategy to overcome the mentioned shortcomings of the canonical MFO algorithm. The moth memory mechanism is inspired by moths’ behavior in nature in remembering their experiences [118], which is defined by Definition 1. Moreover, the AWAS strategy is introduced in Definition 2, to possibly escape the trapped moths from the local optima and alleviate the premature convergence. The pseudo-code and the flowchart of the proposed I-MFO are shown in Algorithm 1 and Figure 2, respectively.

**Definition** **1** **(Moth** **memory** **construction).** *Suppose Mem = {Mem_1_, Mem_2_, …, Mem_i_, …, Mem_N_} is finite set of N moths’ memories. The moth memory M_i_ is denoted by Mem_i_ = (Mbest_i_, Fbest_i_), where Mbest_i_ is the best position of M_i_ obtained so far, and Fbest_i_ is the fitness value of Mbest_i_. In the first iteration t, the best position Mbest_i_ (t = 1) ← M_i_ (t = 1) and Fbest_i_ (t = 1) ← OM_i_ (t = 1). For the rest of the iterations, Mbest_i_ (t > 1) ← M_i_ (P) and Fbest_i_ (t > 1) ← OM_i_ (P) such that {OM_i_ (P) < Fbest_i_, P = 2, …, t}. The moth memory construction is formulated in Equation (4).*

(4) $$If\;\;t=1\;\mathrm{then}\;Fbest_{i}\left(t\right)\leftarrow OM_{i}\left(t\right)\;and\;Mbest_{i}\left(t\right)\leftarrow M_{i}\left(t\right)\;\begin{array}{r} If\;\;t>1\;\mathrm{then}\;Fbest_{i}\left(t\right)\leftarrow OM_{i}\left(P\right)\;and\;Mbest_{i}\left(t\right)\leftarrow M_{i}\left(P\right),\\ Such\;that\;\;∄\;s\;\in \left\{2,\;\ldots ,\;t\right\},\;OM_{i}\left(s\right)<OM_{i}\left(P\right) \end{array}$$

**Algorithm 1.** The pseudo-code of I-MFO
**Algorithm of improved moth-flame optimization (I-MFO)**
**Input:** Maximum iterations (*MaxIt*), Number of moths (*N*), and Dimension size (*D*).**Output:** The best flame position and its fitness value.1
**Begin**
2  Randomly distributing *M* moths in the D-dimensional search space.3  Calculating moths’ fitness (*OM*).4  **Set** *t* = 1.5  *OF* ← sort (*OM*).6  *F*  ← sort (*M*).7  Defining the moth memory *Mbest* and *Fbest* using Definition 1.8   **While** *t* ≤ *MaxIt*9    Updating *F* and *OF* by the best *N* moths from *F* and current *M*.10    Updating *flame_no* using Equation (3)11    **For**
*i* = 1: *N*12      Computing the distance between moth *M_i_* (t) and flame *F_j_* (t) using Equation (2).13      Updating the position of *M_i_* (t) using Equation (1).14      Computing the fitness value of *M_i_* (t) and update *OM_i_* (t).15      **If** *Fbest_i_* (t) < *OM_i_* (t) 16        Selecting a random moth *M_r_* (t).17        Updating the position of *M_i_* (t) using AWAS defined in Definition 2.18        Updating the fitness value *OM_i_* (t).19      
**End if**
20     Updating the moth memory *M_i_* using Definition 1. 21    
**End for**
22    Updating the position and fitness value of the global best flame.23    *t* = *t* + 1.24   **End while**

**Definition** **2**  (AWAS strategy)**.** *Consider TM (t) = {M_1_, …, M_i_, …} as a finite set of moths trapped in the current iteration t such that M_i_ could not dominate its Mem_i_ (OM_i_ (t) > Fbest_i_). Then, to possibly free the trapped moth M_i_ (t + 1) from the local optimum, its new position is computed by Equation (5), where F_gbest j_ (t) is the jth dimension of the global best flame, r_i_ is a random number between interval (0, 1), M_rj_ (t) is the value of a random moth position. The flight length fl_i_ (t) for moth M_i_ is computed by Equation (6), where δ_1_ and δ_2_ are defined by the user, NF is the number of flights determined randomly in [1, D], and q is the current flight number. In fact, using AWAS strategy with the random NF provides advantage through which the trapped moth M_i_ can be moved to a better position*.

(5) $$M_{ij}\;\left(t+1\right)=F_{gbest}{}_{j}\;\left(t\right)+r_{i}\times fl_{i}\;\left(t\right)\times \left(M_{rj}\;\left(t\right)-M_{ij}\;\left(t\right)\right)$$

(6) $$fl_{i}\;\left(t\right)=\delta_{1}-q\times \left(\frac{\delta_{2}}{NF}\right)\;$$

## 4. Numerical Experiment and Analysis

In this section, the performance of the proposed I-MFO has been evaluated using the CEC 2018 [102] benchmark. Moreover, the proposed algorithm was compared with the state-of-the-art metaheuristic algorithms including SA [37], CGA [42], GWO [58], WOA [67], ChOA [59], AOA [55], canonical MFO [74], and its variants such as LMFO [92], WCMFO [95], and SMFO [96]. The parameter settings of comparative algorithms are adjusted as in their original papers and are reported in Table 1. The obtained results are reported in Table 2, Table 3 and Table 4, where the bold values show the winner algorithm. Furthermore, at the end of each table, the symbols W, T, and L demonstrate the number of wins, ties, and losses of each algorithm, respectively.

### 4.1. Benchmark Test Functions and Experimental Environment

The performance of the proposed algorithm is evaluated using the CEC 2018 benchmark functions with various dimensions of 30, 50, and 100. This benchmark contains 29 test functions with a diverse set of characteristics: unimodal, simple multimodal, hybrid, and composition. Test functions F_1_ and F_3_ are unimodal functions and they are adequate for evaluating the exploitation of algorithms. Test functions F_4_–F_10_ are multimodal with many local optima which are suitable to assess the exploration abilities of algorithms. Test functions F_11_–F_20_ are hybrid and F_21_–F_30_ are composition functions that can evaluate the local optima avoidance ability and balance between exploration and exploitation.

Due to the randomization of SI algorithms and to guarantee that the comparisons are fair, all experiments for each function are repeated 30 times separately on a laptop with characteristics: Intel Core i7-10750H CPU (2.60 GHz) and 24 GB of memory. The MATLAB programming language version R2020a and Windows 10 operating system were used to conduct all experiments. All algorithms were run under the same conditions, with the population size (*N*) 100 and the maximum number of iterations (*MaxIt*) (*D* × 10^4^)/*N*.

### 4.2. Exploitation and Exploration Analysis

In this experimental evaluation, the unimodal functions F_1_ and F_3_ are considered to assess the exploitation abilities, while the multimodal test functions F_4_–F_10_ are dedicated to evaluating the exploration capabilities.

**Figure 3:**
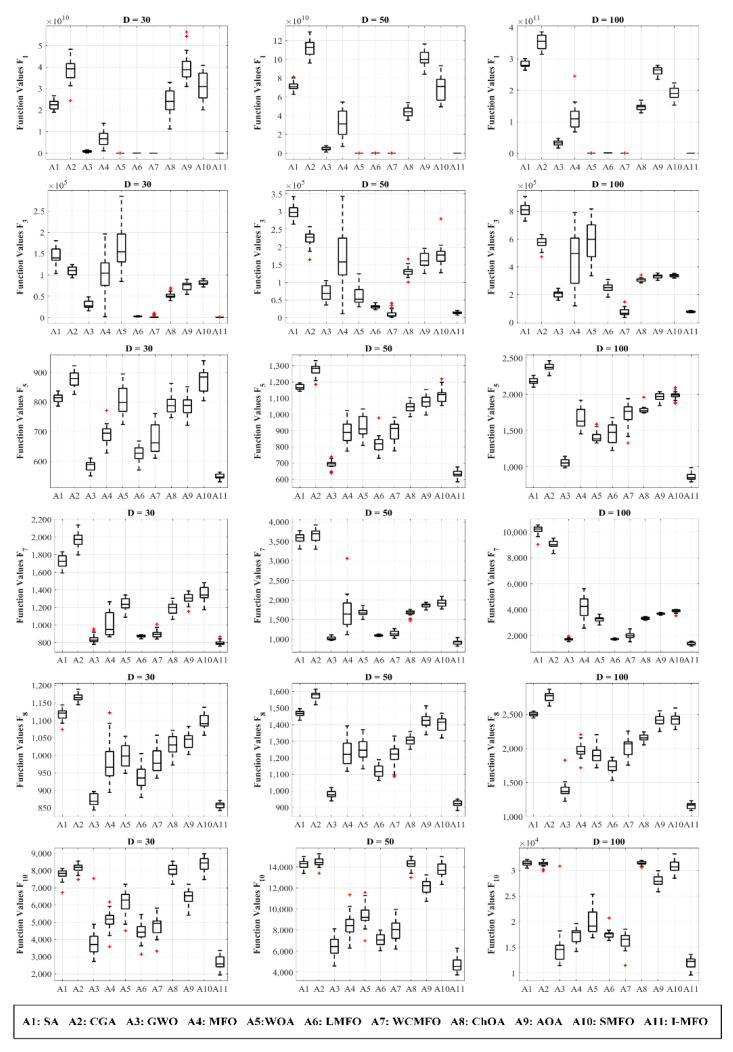
The comparison of fitness distribution on unimodal and multimodal functions.

According to Table 2, the results prove that the I-MFO provides superior exploitation abilities around the optimum solution for all dimensions, particularly on test function F_1_. The results of multimodal test functions are evidence that the exploration capability of the I-MFO significantly outperforms all other algorithms in different dimensions. The comparison and reported results lead to the conclusion that the proposed I-MFO has an effective exploration ability due to the randomness movements of trapped moths by the AWAS strategy. Meanwhile, considering the best flame position and limited value of flight length (*fl*) causes the exploitation ability to remain functional in the course of iterations. Moreover, the comparison of fitness distribution is shown by box and whiskers diagrams in Figure 3. The diagrams predominately demonstrate that the proposed I-MFO can find the best solutions during the optimization process, which verifies that its exploration and exploitation abilities are more sufficient than other competitors. 

### 4.3. Local Optima Avoidance Evaluation

**Figure 4:**
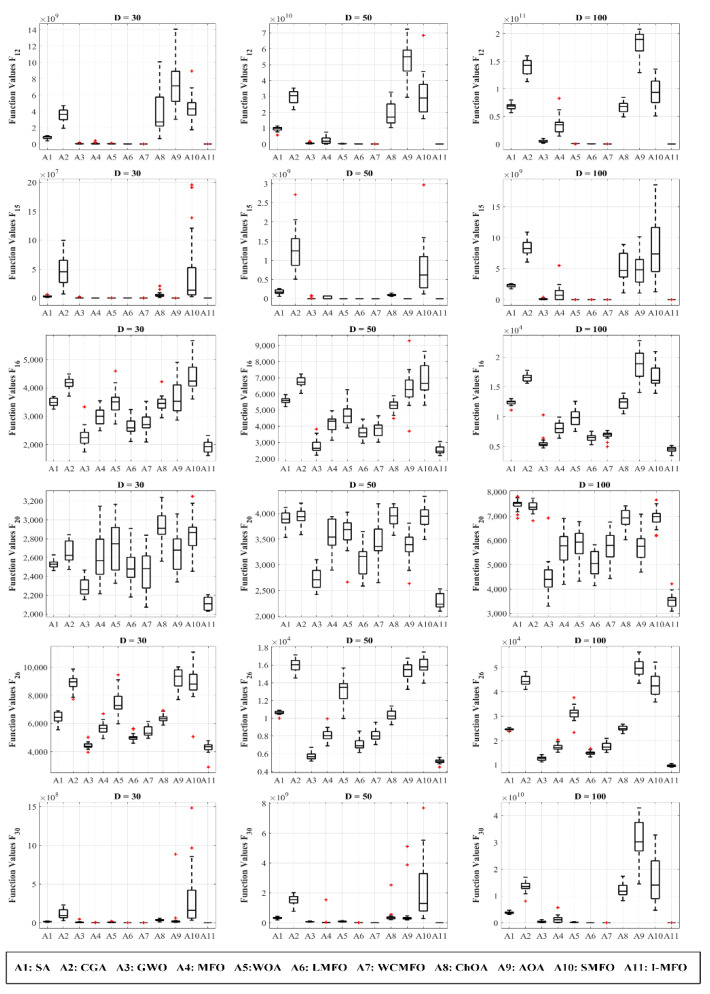
The comparison of fitness distribution on hybrid and composition functions.

This experimental evaluation is benchmarking the ability of the proposed algorithm against the contender algorithms in terms of local optima avoidance and striking a balance between exploration and exploitation by considering hybrid and composition function results. The obtained results tabulated in Table 3 and Table 4 indicate that the proposed I-MFO algorithm is superior to the contender algorithms in dimensions 30, 50, and 100. The main reason is that the AWAS strategy helps trapped moths to escape the local optima and obtain a better position by changing random dimensions of trapped moths with dimensions of the best flame and a random moth’s position. The random moth causes the trapped moth to explore the search space and increases the population diversity while considering the best flame enhances the exploitation capabilities of the algorithm simultaneously. Furthermore, Figure 4 visualizes the comparison of fitness distribution using box and whiskers diagrams in which almost all diagrams demonstrate that the proposed I-MFO can find the best solutions during the optimization process. It verifies that I-MFO can provide satisfactory equilibration between exploration and exploitation.

### 4.4. I-MFO Overall Effectiveness

The overall effectiveness (OE) [50] of the I-MFO and other contender algorithms is computed by using results reported in Table 2, Table 3 and Table 4. The *OE* results tabulated in Table 5 are calculated using Equation (7), where *N* indicates the number of test functions and *L* is the number of losses of each algorithm. The results reveal that I-MFO with overall effectiveness of 92% is the most effective algorithm for all dimensions: 30, 50, and 100.

(7) $$OE\;\left(\%\right)=\left(\frac{N-L}{N\;}\right)\times 100$$

### 4.5. Convergence Behavior Analysis

**Figure 5:**
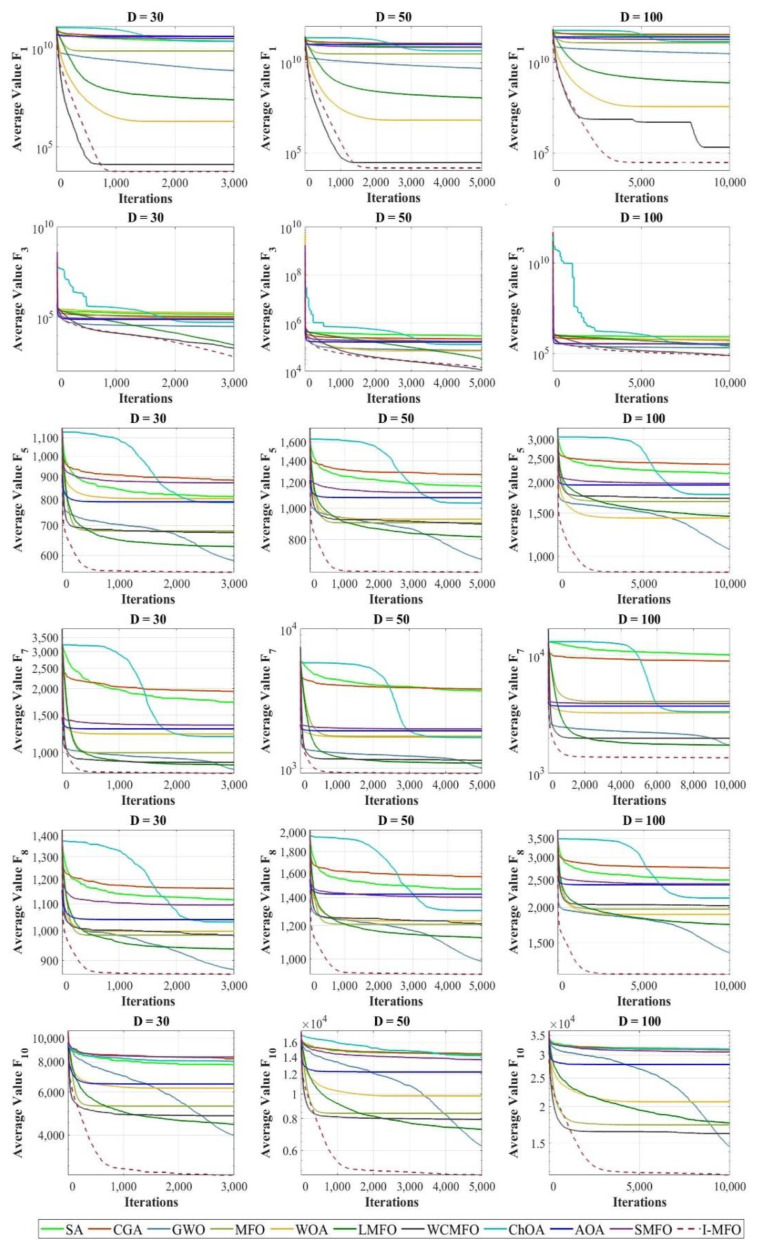
The convergence curves of algorithms in unimodal and multimodal test functions.

**Figure 6:**
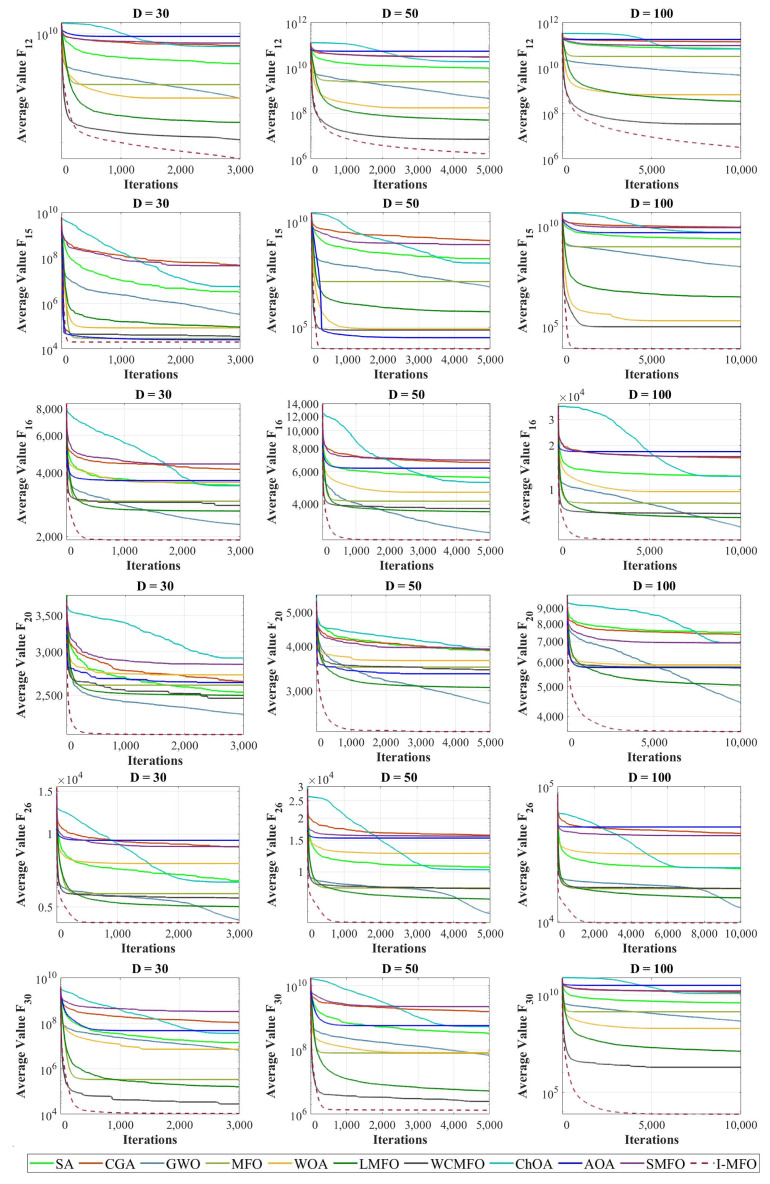
The convergence curves of algorithms in hybrid and composition test functions.

In this section, the convergence behavior of I-MFO is assessed and compared with contender algorithms on some selected functions with dimensions 30, 50, and 100. The convergence curves of the best fitness values obtained by each algorithm on unimodal and multimodal test functions are plotted in Figure 5. Moreover, the convergence curves of hybrid and composition test functions are plotted in Figure 6. 

Investigating convergence behaviors of the I-MFO reveals that it shows various convergence behaviors. The most common behavior is an accelerated descent with the fastest accurate solutions toward the promising area in the early iterations, which can be seen in 30D (F_5_, F_15_, F_16_, F_26_), 50D (F_6_, F_15_, F_16_, F_26_, F_30_), and 100D (F_5_, F_7_, F_8_, F_15_, F_16_, F_26_). For some functions such as 30D (F_1_, F_7_, F_8_, F_18_, F_30_), 50D (F_1_, F_8_, F_18_), and 100D (F_1_, F_10_, F_18_, F_30_), the I-MFO shows abrupt movements in the first half of iterations and very low variations for the second half, which proves the efficient balance between exploration and exploitation. Finally, for 30D (F_3_, F_7_, F_10_, F_12_ F_20_), 50D (F_3_, F_5_, F_8_, F_10_, F_12_, F_18_, F_20_), and 100D (F_3_, F_12_, F_18_, F_20_), the I-MFO starts its convergence with a steep descent slope and then changes to a gradual trend toward the optimum solutions until final iterations. This behavior demonstrates the ability of the I-MFO in escaping from the local optimum and taking advantage of the last iterations.

### 4.6. Population Diversity Analysis

In metaheuristic algorithms, the population diversity maintenance is important throughout the optimization process. The low diversity among search agents may cause the algorithm to plunge into the local optimum. In this experiment, the population diversity of the proposed I-MFO and contender algorithms is measured by a moment of inertia (*I_c_*) [119], where the *I_c_* is the spreading of each individual from their mass center given by Equation (8) and the mass center *c_j_* for *j* = 1, 2 … *D* is calculated by Equation (9).

(8) $$I_{c}=\overset{N}{\underset{i=1}{\sum }}\overset{D}{\underset{j=1}{\sum }}{\left(M_{ij}-c_{j}\right)}^{2}$$

(9) $$c_{j}=\frac{1}{D}\;\overset{N}{\underset{i=1}{\sum }}M_{ij}$$

**Figure 7:**
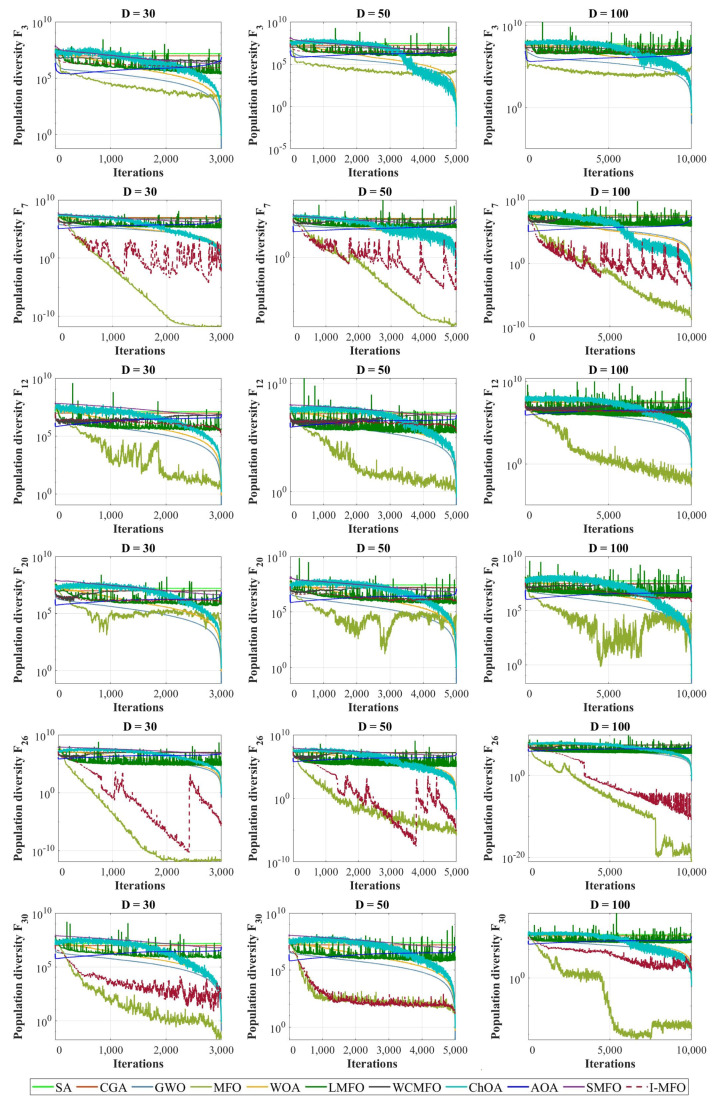
The population diversity of algorithms in different test functions.

**Figure 8:**
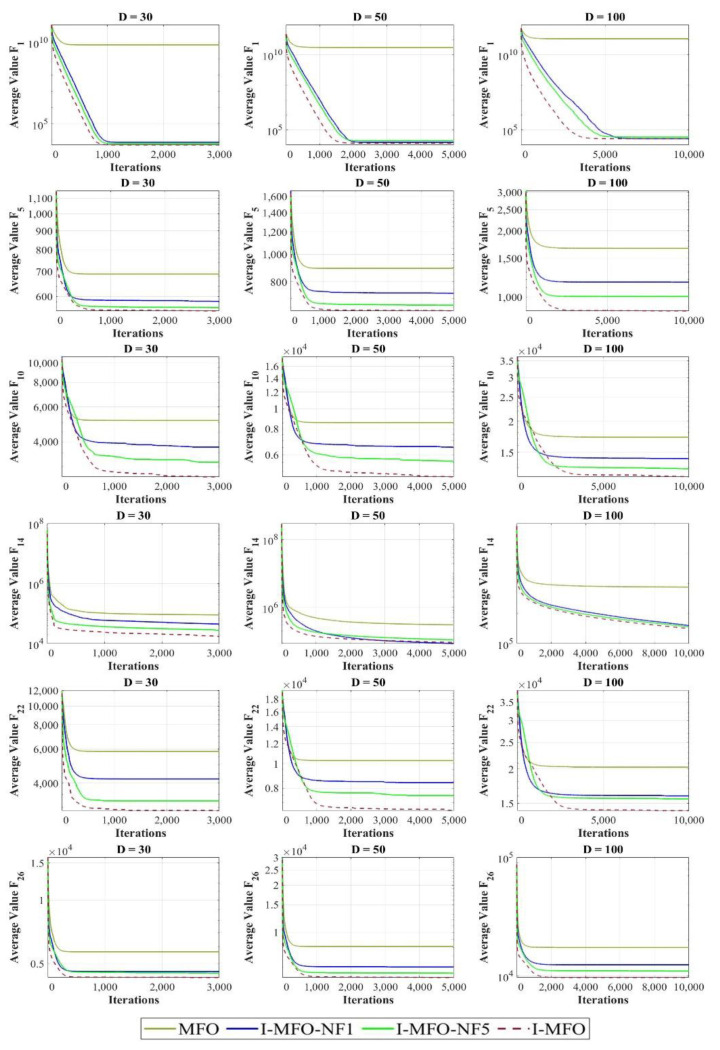
The sensitivity analysis on the *NF* parameter.

The presented population diversity measures the distribution of search agents, and the diversity’s changing slope for the proposed algorithm and contender algorithms is plotted in Figure 7. This experiment is conducted on some CEC 2018 benchmark functions with dimensions 30, 50, and 100. Comparing the convergence curves in Figure 5 and Figure 6 and the plotted diversity in Figure 8 reveals that I-MFO can effectively maintain diversification among solutions until the near-optimal solution is met.

### 4.7. Sensitivity Analysis on the Number of Flight (NF) Parameter

As discussed in Definition 2, the *NF* parameter is the number of opportunities for each trapped moth to fly in the search space and possibly obtain a better position. Hence, in this experiment, the impact of considering different values for the *NF* parameter is evaluated and discussed. The plotted curves in Figure 8 illustrate the convergence behavior of the MFO compared with different variants of I-MFO algorithm. In I-MFO-NF1 and I-MFO-NF5, the value of *NF* is considered by 1 and 5 while in I-MFO, the value of *NF* is set by a random number in [1, *D*]. The results gained for different dimensions 30, 50, and 100 reveal that setting *NF* by a random number limited by the dimension has an advantage for trapped moths to possibly escape from the local optima for different test functions.

### 4.8. Impact Analysis of Applying AWAS Strategy

In this experiment, the impact of applying the AWAS strategy is analyzed on some selected functions of the CEC 2018 benchmark for different dimensions 30, 50, and 100. The proposed AWAS strategy can ameliorate the MFO’s weaknesses described in Section 2. To adequately assess the impact of applying the AWAS strategy, in this experiment we consider MFO, I-MFO, and its three variations including I-MFO-10%, I-MFO-40%, and I-MFO-80% which indicate the percentage of trapped moths that are randomly selected to possibly escape from the local optima using the proposed AWAS strategy. 

**Figure 9:**
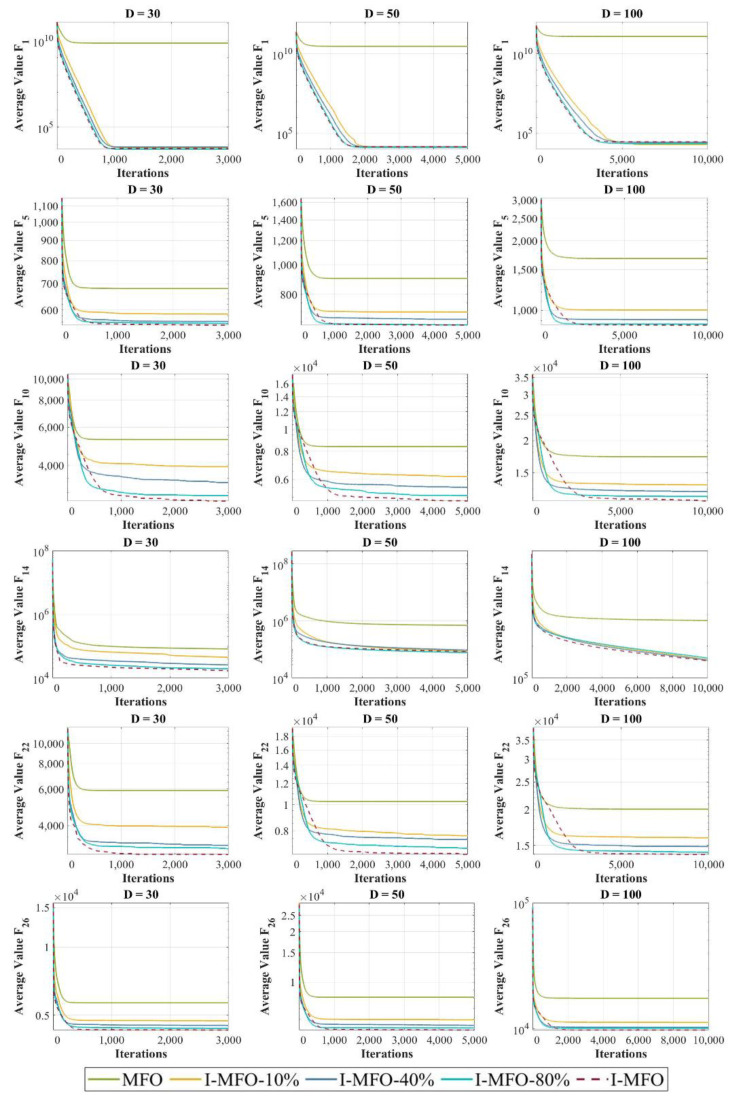
Impact analysis of applying AWAS strategy on different percentages of trapped moths.

The first row of Figure 9 indicates convergence curves for unimodal F_1_, where the I-MFO and its variations outperform the MFO for all dimensions. Specifically, for dimension 100, the I-MFO-10% offers superior outcomes while it has less computational cost compared to the I-MFO. The curves provided for multimodal F_5_ and F_10_ indicate that the I-MFO offers better solutions, while in the next ranks, I-MFO-80%, I-MFO-40%, and I-MFO-10% outperform the MFO. The hybrid test function is shown in the fourth row of Figure 9, where the I-MFO and its variations keep converging toward the global optimum with a steep slope until the final iterations. The I-MFO and its variations can also find better solutions for the composition functions F_22_ and F_26_, wherein for these functions, as the number of dimensions grows, the significance of the AWAS strategy in guiding the population toward the global optimum region and avoiding local optima entrapment becomes clearer. Although the provided results demonstrate that the I-MFO with 100% of trapped moths applied to AWAS strategy mostly provides better solutions for different dimensions and search spaces, other variations of the I-MFO can also provide competitive performance while they have the advantage of lower computational cost compared to the I-MFO.

## 5. Statistical Analysis

In this section, the results obtained in the preceding section are first statistically analyzed using the non-parametric Friedman test. The Bonferroni and Tukey post hoc producers are then conducted to establish proper comparisons between the proposed algorithm and comparative algorithms.

### 5.1. Non-Parametric Friedman Test

The Friedman test is performed to rank the significance of the superiority algorithms statistically [120,121]. The obtained results for unimodal and multimodal test functions are tabulated in Table 6 and the results for hybrid and composition functions are reported in Table 7. This statistical analysis shows that the I-MFO is first rank on all test functions for dimensions of 30, 50, and 100.

**Figure 10:**
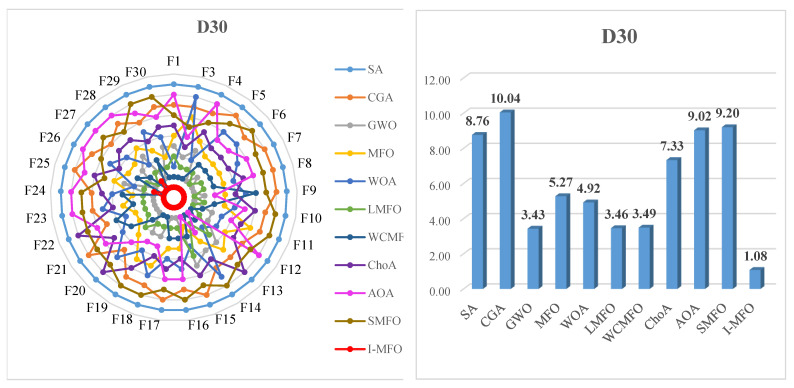

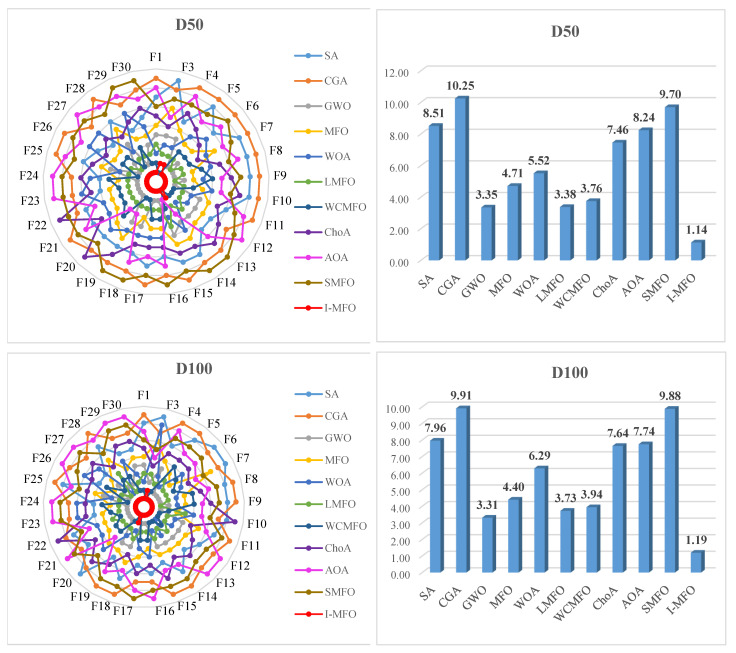
The radar graphs and bar charts of algorithms in different dimensions.

Figure 10 contains six charts to visually show the ranking of the I-MFO and contender algorithms in different dimensions. The left side illustrates the ranking of algorithms in various functions of the CEC 2018 benchmark, while the right side shows the bar chart of Friedman test average results. The radar graph shows that the I-MFO outperforms other algorithms in different dimensions as the smaller size of the I-MFO indicates its first and second rank for all functions. The bar chart provided on the right side reveals that the I-MFO is superior to other comparative algorithms as it has the shortest bar in various dimensions of 30, 50, and 100.

### 5.2. Post Hoc Analysis

In the post hoc analysis [120], we evaluated the proposed hypothesis between the control method and the rest of the compared methods in Table 8 by employing Bonferroni and Tukey’s multiple comparison producers. In this experiment, the level of significance is *α* = 0.05, which determines whether or not a hypothesis is acceptable by comparing the significant difference (*p*-value) between each pair of algorithms. Since gained *p*-values for all dimensions 30, 50, and 100 are less than *α* = 0.05, it reveals that there are significant differences between the performances of the I-MFO and other compared algorithms.

## 6. Applicability of I-MFO Algorithm to Solve Mechanical Engineering Problems

In this section, three constrained mechanical engineering problems from the latest test-suite CEC 2020 [103] are considered to evaluate the applicability of the I-MFO algorithm in real-world applications. To achieve a fair comparison, the algorithms were run 20 times with the population size (*N*) 20 and maximum iterations (*MaxIt*) (*D* × 10^4^)/*N*. In this experimental evaluation, the proposed algorithm and contender algorithms compete to solve three different problems that consist of a gas transmission compressor design problem, three-bar truss, and tension/compression spring design.

P_1_: Gas transmission compressor design problem

**Figure 11:**
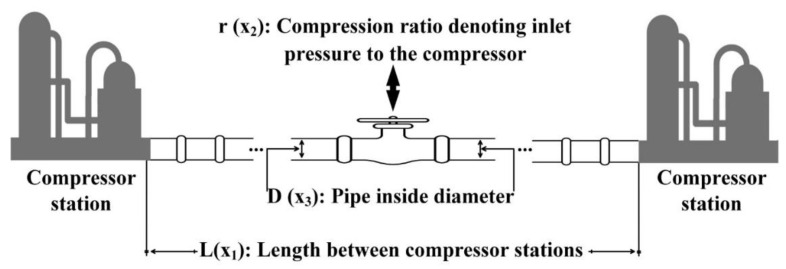
Gas transmission compressor design problem.

Minimization of the objective function using four design variables is the main goal of the gas transmission compressor design problem. This problem is illustrated and formulated in Figure 11 and Equation (10). The performance of the proposed algorithm is evaluated against the contender algorithms to solve this problem and the obtained results are tabulated in Table 9. As shown in this table, the I-MFO is superior in addressing this issue.

(10) $$\begin{array}{l} \mathrm{Minimize}\;f\left(\bar{x}\right)=8.61\times 10^{5}x_{1}^{\frac{1}{2}}x_{2}x_{3}^{-\frac{2}{3}}x_{4}^{-\frac{1}{2}}+\left(3.69\right)\times 10^{4}x_{3}+\left(7.72\right)\times 10^{8}x_{1}^{-1}\;x_{2}^{0.219}-\left(765.43\right)\times 10^{6}x_{1}^{-1}\\ \mathrm{Subject}\;\mathrm{to}\;x_{4}x_{2}^{-2}+x_{2}^{-2}-1\leq 0\\ \mathrm{Variable}\;\mathrm{range}\;20\leq x_{1}\leq 50,\;1\leq x_{2}\leq 10,\;20\leq x_{3}\leq 45,\;0.1\leq x_{4}\leq 60 \end{array}$$

P_2_: Three-bar truss problem

**Figure 12:**
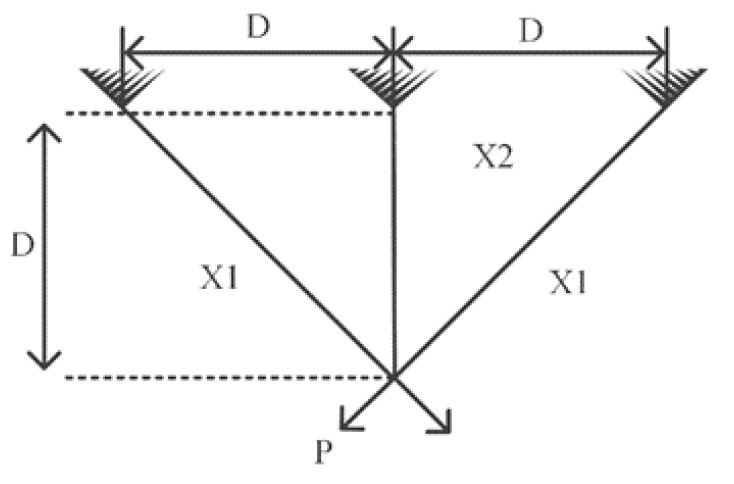
Three-bar truss problem.

In this problem, three constraints and two variables are utilized to formulate the objective function, which is the weight of the bar structures. The schematic and formulation of this problem are represented in Figure 12 and Equation (11), respectively. The proposed I-MFO algorithm and comparative algorithms are compared for solving this problem. The attained results from this experiment are tabulated in Table 10, in which the I-MFO algorithm outperforms other algorithms in approximating the optimal values for variables with minimum weight.

(11) $$\begin{array}{l} \mathrm{Minimize}\;f\left(x\right)=l\times \left(x_{2}+2\sqrt{2}\;x_{1}\right)\\ \begin{array}{l} \mathrm{Subject}\;\mathrm{to}\;g_{1}\left(x\right)=\frac{x_{2}}{2x_{2}x_{1}+\sqrt{2}\;x_{1}^{2}}\;p-\sigma \leq 0\\ \;\;\;\;\;\;\;\;\;\;\;\;\;\;\;\;\;\;\;\;\;g_{2}\left(x\right)=\frac{x_{2}+\sqrt{2}\;x_{1}}{2x_{2}x_{1}}\;p-\sigma \;\leq 0\\ \;\;\;\;\;\;\;\;\;\;\;\;\;\;\;\;\;\;\;\;\;g_{3}\left(x\right)=\frac{1}{x_{1}+\sqrt{2}\;x_{2}}\;p-\sigma \leq 0 \end{array}\\ \mathrm{where}\;\;\;\;\;\;\;\;\;\;\;\;\;\;l=100\;\mathrm{cm},\;p=\frac{2KN}{cm^{2}},\;\mathrm{and}\;\sigma =\frac{2KN}{cm^{2}}\\ \mathrm{Variable}\;\mathrm{range}\;0\;\leq x_{1}\;\leq 1\\ \;\;\;\;\;\;\;\;\;\;\;\;\;\;\;\;\;\;\;\;\;0\;\leq x_{2}\;\leq 1 \end{array}$$

P_3_: Tension/compression spring design problem

**Figure 13:**
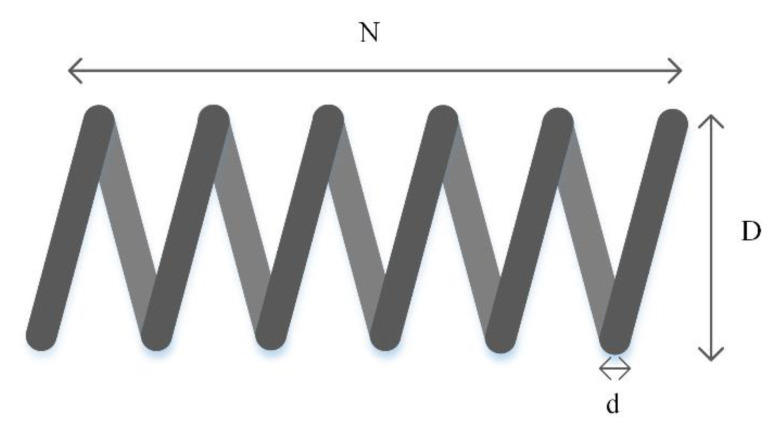
Tension/compression spring design problem.

In the tension/compression spring design problem, the objective is to minimize the weight of the tension/compression spring by considering three variables and four constraints. As shown in Figure 13, the variables are wire diameter (*d*), the number of active coils (*N*), and mean coil diameter (*D*). The problem and its constraints are described in Equation (12) and results are reported in Table 11.

(12) $$\begin{array}{l} \mathrm{Minimize}\;f\left(x\right)=x_{1}^{2}x_{2}\;\left(2+x_{3}\right)\\ \begin{array}{l} \mathrm{Subject}\;\mathrm{to}\;g_{1}\left(x\right)=1-\;\frac{x_{2}^{3}x_{3}}{71,785x_{1}^{4}}\;\leq 0\\ \;\;\;\;\;\;\;\;\;\;\;\;\;\;\;\;\;\;\;\;\;g_{2}\left(x\right)=\;\frac{4x_{2}^{2}-x_{1}x_{2}}{12,566\left(x_{2}x_{1}^{3}-x_{1}^{4}\right)}+\;\frac{1}{5108x_{1}^{2}}-1\;\leq 0\\ \;\;\;\;\;\;\;\;\;\;\;\;\;\;\;\;\;\;\;\;\;g_{3}\left(x\right)=1-\;\frac{140.45x_{1}}{x_{2}^{2}x_{3}}\;\leq 0\\ \;\;\;\;\;\;\;\;\;\;\;\;\;\;\;\;\;\;\;\;\;g_{4}\left(x\right)=\;\frac{x_{1}+x_{2}}{1.5}-1\;\leq 0 \end{array}\\ \mathrm{Variable}\;\mathrm{range}\;0.05\;\leq x_{1}\;\leq 2.00\\ \;\;\;\;\;\;\;\;\;\;\;\;\;\;\;\;\;\;\;\;\;0.25\;\leq x_{2}\;\leq 1.30\\ \;\;\;\;\;\;\;\;\;\;\;\;\;\;\;\;\;\;\;\;\;2.00\;\leq x_{3}\;\leq 15.0 \end{array}$$

The results of the mechanical engineering problems tabulated in Table 9, Table 10 and Table 11 demonstrate the fact that the I-MFO is superior to other algorithms for solving real-world mechanical engineering problems.

## 7. Conclusions and Future Works

The transverse orientation behavior of moths while encountering artificial lights is the main inspiration behind the MFO algorithm to successfully solve optimization problems. However, as with most of the SI algorithms, the MFO suffers from premature convergence, local optima entrapping, low population diversity, and imbalance between exploration and exploitation. These drawbacks make the MFO uncompetitive in solving complex and real-world optimization problems. Therefore, an improved version of the MFO named I-MFO is proposed to improve the MFO algorithm from the perspective of alleviating premature convergence, maintaining population diversity, avoiding local optima trapping, and striking a balance between exploration and exploitation.

To detect local optima-trapped moths, a memory mechanism is defined for each moth. Then, the adapted wandering around search (AWAS) strategy is introduced to possibly free detected trapped moths from local optima by changing their positions while considering the best flame and a random moth position. The CEC 2018 benchmark tasks were conducted to evaluate the performance of the I-MFO, where the reported results in Table 2, Table 3 and Table 4 and OE in Table 5 prove I-MFO’s superior performance over 92% of test functions. The multimodal test function results reported in Table 2 are clear evidence of the fact that the I-MFO boosts exploration rate, especially in more complex problems. The hybrid and composition test function results tabulated in Table 3 and Table 4 support the claim that the proposed I-MFO enhances the balance between exploration and exploitation, by which the I-MFO can get out of local optima effectively. The convergence curves also show the local optima avoidance ability and enhanced balance between exploration and exploitation. Moreover, it can be deduced from the population diversity plots that the I-MFO successfully maintains population diversity until a near-optimal solution emerges. 

The sensitivity of the AWAS strategy and *NF* parameter is evaluated on some CEC 2018 benchmark functions for different dimensions, where the results reveal that although the I-MFO offers better solutions in most test functions, other variations of the I-MFO can also provide competitive outcomes for some functions and dimensions. The statistical efficiency of the I-MFO is investigated by the Friedman test and post-hoc analysis, which revealed that the proposed I-MFO outperforms other contender algorithms for various test functions. In the end, the outcomes of the mechanical engineering problems from the latest test-suite CEC 2020 demonstrate that the proposed I-MFO is applicable for solving real-world mechanical engineering problems. Although I-MFO provides competitive results for solving global optimization and engineering tasks, like most improvements, it consumes more time compared to the canonical MFO because it uses the AWAS strategy. Hence, in practice, the I-MFO may not be suitable for solving large-scale real-time problems. For future works, a multi-objective version of I-MFO can be developed for solving continuous multi-objective problems. Moreover, extending I-MFO to the discrete version for solving discrete optimization tasks such as the community detection problem is a worthwhile direction.

## Figures and Tables

**Table 1:** Parameter settings of the I-MFO and other contender algorithms.

Algorithms	Parameter Settings
SA	*T*_0_ = 10.
CGA	*IPMut* = 0.9, *PXcross* = 0.5.
GWO	The parameter *a* is linearly decreased from 2 to 0.
MFO	*b* = 1, *a* is decreased linearly from −1 to −2.
WOA	*α* variable decreases linearly from 2 to 0, *b* = 1.
LMFO	*β* = 1.5, *µ* and *v* are normal distributions, *Γ* is the gamma function.
WCMFO	The number of rivers and sea = 4.
ChOA	*f* decreases linearly from 2 to 0.
AOA	*µ* = 0.5, *α* = 5.
SMFO	*r*_4_ = random number between interval (0, 1).
I-MFO	*δ*_1_ = 2.02, *δ*_2_ = 1.08, *NF* = random number between 1 and *D*.

**Table 2:** Comparison of optimization results obtained from unimodal and multimodal test functions.

F	D	Metrics	SA (1983)	CGA (2000)	GWO (2014)	MFO (2015)	WOA (2016)	LMFO (2016)	WCMFO (2019)	ChOA (2020)	AOA (2021)	SMFO (2021)	I-MFO
F_1_	30	Avg	2.251 × 10^10^	3.794 × 10^10^	8.223 × 10^8^	6.952 × 10^9^	1.906 × 10^6^	2.402 × 10^7^	1.328 × 10^4^	2.238 × 10^10^	3.943 × 10^10^	3.091 × 10^10^	**5.859 × 10^3^**
		Min	1.897 × 10^10^	2.437 × 10^10^	4.404 × 10^7^	1.027 × 10^9^	5.654 × 10^5^	1.731 × 10^7^	1.214 × 10^2^	1.123 × 10^10^	2.791 × 10^10^	2.010 × 10^10^	1.488 × 10^2^
	50	Avg	7.170 × 10^10^	1.126 × 10^11^	4.522 × 10^9^	3.099 × 10^10^	7.172 × 10^6^	1.091 × 10^8^	2.826 × 10^4^	4.407 × 10^10^	9.968 × 10^10^	6.933 × 10^10^	**1.430 × 10^4^**
		Min	6.279 × 10^10^	9.612 × 10^10^	1.231 × 10^9^	7.095 × 10^9^	1.980 × 10^6^	7.284 × 10^7^	6.883 × 10^2^	3.201 × 10^10^	8.424 × 10^10^	4.945 × 10^10^	1.948 × 10^2^
	100	Avg	2.830 × 10^11^	3.547 × 10^11^	3.207 × 10^10^	1.173 × 10^11^	3.677 × 10^7^	7.525 × 10^8^	2.017 × 10^5^	1.457 × 10^11^	2.617 × 10^11^	1.908 × 10^11^	**2.881 × 10^4^**
		Min	2.638 × 10^11^	3.147 × 10^11^	1.634 × 10^10^	6.748 × 10^10^	1.409 × 10^7^	6.332 × 10^8^	1.093 × 10^4^	1.282 × 10^11^	2.343 × 10^11^	1.527 × 10^11^	1.033 × 10^2^
F_3_	30	Avg	1.439 × 10^5^	1.095 × 10^5^	2.993 × 10^4^	1.009 × 10^5^	1.715 × 10^5^	2.786 × 10^3^	1.887 × 10^3^	5.221 × 10^4^	7.488 × 10^4^	8.189 × 10^4^	**6.106 × 10^2^**
		Min	1.029 × 10^5^	9.312 × 10^4^	1.576 × 10^4^	1.920 × 10^3^	8.481 × 10^4^	1.424 × 10^3^	3.092 × 10^2^	3.954 × 10^4^	5.445 × 10^4^	7.186 × 10^4^	3.388 × 10^2^
	50	Avg	2.994 × 10^5^	2.241 × 10^5^	7.147 × 10^4^	1.650 × 10^5^	6.180 × 10^4^	3.151 × 10^4^	**1.150 × 10^4^**	1.306 × 10^5^	1.648 × 10^5^	1.775 × 10^5^	1.460 × 10^4^
		Min	2.647 × 10^5^	1.648 × 10^5^	3.628 × 10^4^	1.176 × 10^4^	3.098 × 10^4^	2.291 × 10^4^	7.428 × 10^2^	1.006 × 10^5^	1.249 × 10^5^	1.273 × 10^5^	7.827 × 10^3^
	100	Avg	8.110 × 10^5^	5.742 × 10^5^	2.023 × 10^5^	4.556 × 10^5^	5.928 × 10^5^	2.495 × 10^5^	**7.361 × 10^4^**	3.071 × 10^5^	3.330 × 10^5^	3.366 × 10^5^	7.625 × 10^4^
		Min	7.298 × 10^5^	4.725 × 10^5^	1.595 × 10^5^	1.191 × 10^5^	3.355 × 10^5^	1.819 × 10^5^	3.430 × 10^4^	2.701 × 10^5^	3.027 × 10^5^	3.182 × 10^5^	6.767 × 10^4^
F_4_	30	Avg	1.587 × 10^3^	7.327 × 10^3^	5.441 × 10^2^	9.082 × 10^2^	5.476 × 10^2^	4.928 × 10^2^	4.886 × 10^2^	2.971 × 10^3^	8.808 × 10^3^	5.977 × 10^3^	**4.868 × 10^2^**
		Min	1.354 × 10^3^	6.137 × 10^3^	4.963 × 10^2^	5.424 × 10^2^	4.995 × 10^2^	4.755 × 10^2^	4.239 × 10^2^	1.134 × 10^3^	3.824 × 10^3^	3.030 × 10^3^	4.704 × 10^2^
	50	Avg	7.832 × 10^3^	2.608 × 10^4^	8.767 × 10^2^	4.097 × 10^3^	6.676 × 10^2^	5.907 × 10^2^	**5.493 × 10^2^**	9.176 × 10^3^	2.582 × 10^4^	1.879 × 10^4^	5.550 × 10^2^
		Min	6.386 × 10^3^	1.598 × 10^4^	6.745 × 10^2^	1.216 × 10^3^	5.138 × 10^2^	5.084 × 10^2^	4.849 × 10^2^	5.017 × 10^3^	1.686 × 10^4^	1.005 × 10^4^	4.210 × 10^2^
	100	Avg	4.778 × 10^4^	1.033 × 10^5^	2.812 × 10^3^	2.348 × 10^4^	9.992 × 10^2^	7.215 × 10^2^	6.423 × 10^2^	2.760 × 10^4^	7.703 × 10^4^	5.544 × 10^4^	**6.318 × 10^2^**
		Min	3.845 × 10^4^	8.305 × 10^4^	1.870 × 10^3^	6.742 × 10^3^	8.615 × 10^2^	6.726 × 10^2^	5.980 × 10^2^	2.116 × 10^4^	6.019 × 10^4^	3.485 × 10^4^	5.772 × 10^2^
F_5_	30	Avg	8.120 × 10^2^	8.777 × 10^2^	5.855 × 10^2^	6.894 × 10^2^	8.044 × 10^2^	6.278 × 10^2^	6.744 × 10^2^	7.877 × 10^2^	7.905 × 10^2^	8.721 × 10^2^	**5.499 × 10^2^**
		Min	7.865 × 10^2^	8.259 × 10^2^	5.508 × 10^2^	6.280 × 10^2^	7.242 × 10^2^	5.707 × 10^2^	6.104 × 10^2^	7.471 × 10^2^	7.217 × 10^2^	8.041 × 10^2^	5.308 × 10^2^
	50	Avg	1.167 × 10^3^	1.273 × 10^3^	6.892 × 10^2^	8.934 × 10^2^	9.209 × 10^2^	8.152 × 10^2^	8.940 × 10^2^	1.037 × 10^3^	1.078 × 10^3^	1.118 × 10^3^	**6.348 × 10^2^**
		Min	1.142 × 10^3^	1.184 × 10^3^	6.379 × 10^2^	7.731 × 10^2^	8.081 × 10^2^	7.287 × 10^2^	7.743 × 10^2^	9.853 × 10^2^	9.951 × 10^2^	1.053 × 10^3^	5.836 × 10^2^
	100	Avg	2.180 × 10^3^	2.374 × 10^3^	1.058 × 10^3^	1.666 × 10^3^	1.413 × 10^3^	1.456 × 10^3^	1.726 × 10^3^	1.789 × 10^3^	1.955 × 10^3^	1.985 × 10^3^	**8.613 × 10^2^**
		Min	2.098 × 10^3^	2.255 × 10^3^	9.864 × 10^2^	1.455 × 10^3^	1.329 × 10^3^	1.226 × 10^3^	1.328 × 10^3^	1.724 × 10^3^	1.842 × 10^3^	1.875 × 10^3^	7.885 × 10^2^
F_6_	30	Avg	6.582 × 10^2^	6.734 × 10^2^	6.043 × 10^2^	6.267 × 10^2^	6.671 × 10^2^	6.038 × 10^2^	6.225 × 10^2^	6.604 × 10^2^	6.655 × 10^2^	6.830 × 10^2^	**6.000 × 10^2^**
		Min	6.468 × 10^2^	6.612 × 10^2^	6.011 × 10^2^	6.144 × 10^2^	6.410 × 10^2^	6.017 × 10^2^	6.095 × 10^2^	6.386 × 10^2^	6.476 × 10^2^	6.615 × 10^2^	6.000 × 10^2^
	50	Avg	6.835 × 10^2^	6.936 × 10^2^	6.105 × 10^2^	6.437 × 10^2^	6.760 × 10^2^	6.094 × 10^2^	6.400 × 10^2^	6.720 × 10^2^	6.837 × 10^2^	6.890 × 10^2^	**6.000 × 10^2^**
		Min	6.691 × 10^2^	6.784 × 10^2^	6.052 × 10^2^	6.270 × 10^2^	6.638 × 10^2^	6.034 × 10^2^	6.165 × 10^2^	6.608 × 10^2^	6.747 × 10^2^	6.780 × 10^2^	6.000 × 10^2^
	100	Avg	7.210 × 10^2^	7.176 × 10^2^	6.275 × 10^2^	6.648 × 10^2^	6.768 × 10^2^	6.398 × 10^2^	6.664 × 10^2^	6.860 × 10^2^	7.028 × 10^2^	7.030 × 10^2^	**6.000 × 10^2^**
		Min	7.164 × 10^2^	7.143 × 10^2^	6.229 × 10^2^	6.466 × 10^2^	6.676 × 10^2^	6.222 × 10^2^	6.526 × 10^2^	6.761 × 10^2^	6.970 × 10^2^	6.865 × 10^2^	6.000 × 10^2^
F_7_	30	Avg	1.728 × 10^3^	1.965 × 10^3^	8.418 × 10^2^	1.011 × 10^3^	1.238 × 10^3^	8.735 × 10^2^	8.985 × 10^2^	1.190 × 10^3^	1.295 × 10^3^	1.349 × 10^3^	**7.964 × 10^2^**
		Min	1.593 × 10^3^	1.798 × 10^3^	7.801 × 10^2^	8.671 × 10^2^	1.089 × 10^3^	8.438 × 10^2^	8.402 × 10^2^	1.063 × 10^3^	1.154 × 10^3^	1.175 × 10^3^	7.595 × 10^2^
	50	Avg	3.572 × 10^3^	3.656 × 10^3^	1.015 × 10^3^	1.701 × 10^3^	1.684 × 10^3^	1.092 × 10^3^	1.141 × 10^3^	1.663 × 10^3^	1.860 × 10^3^	1.919 × 10^3^	**9.208 × 10^2^**
		Min	3.305 × 10^3^	3.300 × 10^3^	9.654 × 10^2^	1.113 × 10^3^	1.500 × 10^3^	1.065 × 10^3^	1.020 × 10^3^	1.464 × 10^3^	1.744 × 10^3^	1.769 × 10^3^	8.168 × 10^2^
	100	Avg	1.014 × 10^4^	9.052 × 10^3^	1.710 × 10^3^	4.169 × 10^3^	3.250 × 10^3^	1.736 × 10^3^	1.988 × 10^3^	3.320 × 10^3^	3.712 × 10^3^	3.891 × 10^3^	**1.356 × 10^3^**
		Min	9.035 × 10^3^	8.338 × 10^3^	1.542 × 10^3^	2.576 × 10^3^	2.814 × 10^3^	1.649 × 10^3^	1.531 × 10^3^	3.127 × 10^3^	3.579 × 10^3^	3.536 × 10^3^	1.140 × 10^3^
F_8_	30	Avg	1.116 × 10^3^	1.166 × 10^3^	8.713 × 10^2^	9.790 × 10^2^	1.000 × 10^3^	9.379 × 10^2^	9.841 × 10^2^	1.032 × 10^3^	1.041 × 10^3^	1.096 × 10^3^	**8.574 × 10^2^**
		Min	1.074 × 10^3^	1.144 × 10^3^	8.435 × 10^2^	8.938 × 10^2^	9.488 × 10^2^	8.797 × 10^2^	9.344 × 10^2^	9.726 × 10^2^	1.002 × 10^3^	1.058 × 10^3^	8.418 × 10^2^
	50	Avg	1.467 × 10^3^	1.573 × 10^3^	9.792 × 10^2^	1.229 × 10^3^	1.249 × 10^3^	1.119 × 10^3^	1.213 × 10^3^	1.305 × 10^3^	1.426 × 10^3^	1.404 × 10^3^	**9.201 × 10^2^**
		Min	1.427 × 10^3^	1.519 × 10^3^	9.384 × 10^2^	1.118 × 10^3^	1.132 × 10^3^	1.062 × 10^3^	1.087 × 10^3^	1.251 × 10^3^	1.339 × 10^3^	1.320 × 10^3^	8.796 × 10^2^
	100	Avg	2.499 × 10^3^	2.751 × 10^3^	1.397 × 10^3^	1.968 × 10^3^	1.897 × 10^3^	1.740 × 10^3^	2.026 × 10^3^	2.157 × 10^3^	2.404 × 10^3^	2.422 × 10^3^	**1.160 × 10^3^**
		Min	2.448 × 10^3^	2.620 × 10^3^	1.225 × 10^3^	1.717 × 10^3^	1.716 × 10^3^	1.531 × 10^3^	1.756 × 10^3^	2.052 × 10^3^	2.248 × 10^3^	2.276 × 10^3^	1.087 × 10^3^
F_9_	30	Avg	1.079 × 10^4^	1.239 × 10^4^	1.384 × 10^3^	6.278 × 10^3^	7.233 × 10^3^	1.015 × 10^3^	8.747 × 10^3^	6.612 × 10^3^	5.570 × 10^3^	9.591 × 10^3^	**9.882 × 10^2^**
		Min	9.017 × 10^3^	8.805 × 10^3^	1.025 × 10^3^	4.471 × 10^3^	4.425 × 10^3^	9.056 × 10^2^	5.118 × 10^3^	4.627 × 10^3^	4.101 × 10^3^	7.754 × 10^3^	9.065 × 10^2^
	50	Avg	3.302 × 10^4^	4.335 × 10^4^	4.571 × 10^3^	1.644 × 10^4^	1.783 × 10^4^	1.306 × 10^3^	2.195 × 10^4^	2.591 × 10^4^	2.277 × 10^4^	3.066 × 10^4^	**1.305 × 10^3^**
		Min	2.622 × 10^4^	3.603 × 10^4^	2.135 × 10^3^	8.748 × 10^3^	1.187 × 10^4^	9.481 × 10^2^	1.190 × 10^4^	1.969 × 10^4^	1.804 × 10^4^	1.925 × 10^4^	9.853 × 10^2^
	100	Avg	1.197 × 10^5^	1.243 × 10^5^	2.638 × 10^4^	4.507 × 10^4^	3.820 × 10^4^	1.421 × 10^4^	5.208 × 10^4^	6.813 × 10^4^	5.374 × 10^4^	6.959 × 10^4^	**7.081 × 10^3^**
		Min	1.034 × 10^5^	1.079 × 10^5^	1.102 × 10^4^	3.679 × 10^4^	2.557 × 10^4^	3.037 × 10^3^	3.986 × 10^4^	5.806 × 10^4^	4.673 × 10^4^	5.852 × 10^4^	4.038 × 10^3^
F_10_	30	Avg	7.764 × 10^3^	8.149 × 10^3^	3.909 × 10^3^	5.130 × 10^3^	6.156 × 10^3^	4.422 × 10^3^	4.808 × 10^3^	8.037 × 10^3^	6.487 × 10^3^	8.363 × 10^3^	**2.745 × 10^3^**
		Min	6.721 × 10^3^	7.474 × 10^3^	2.718 × 10^3^	3.575 × 10^3^	4.506 × 10^3^	3.149 × 10^3^	3.332 × 10^3^	7.199 × 10^3^	5.410 × 10^3^	7.473 × 10^3^	1.941 × 10^3^
	50	Avg	1.426 × 10^4^	1.446 × 10^4^	6.428 × 10^3^	8.566 × 10^3^	9.478 × 10^3^	7.081 × 10^3^	7.956 × 10^3^	1.419 × 10^4^	1.223 × 10^4^	1.368 × 10^4^	**4.799 × 10^3^**
		Min	1.337 × 10^4^	1.342 × 10^4^	4.582 × 10^3^	6.288 × 10^3^	6.969 × 10^3^	6.045 × 10^3^	6.204 × 10^3^	1.301 × 10^4^	1.058 × 10^4^	1.234 × 10^4^	3.734 × 10^3^
	100	Avg	3.136 × 10^4^	3.121 × 10^4^	1.497 × 10^4^	1.728 × 10^4^	2.012 × 10^4^	1.758 × 10^4^	1.618 × 10^4^	3.141 × 10^4^	2.783 × 10^4^	3.071 × 10^4^	**1.168 × 10^4^**
		Min	3.040 × 10^4^	2.990 × 10^4^	1.141 × 10^4^	1.417 × 10^4^	1.687 × 10^4^	1.631 × 10^4^	1.147 × 10^4^	3.035 × 10^4^	2.582 × 10^4^	2.843 × 10^4^	9.083 × 10^3^
**Ranking**	**30**	**W|T|L**	0/0/9	0/0/9	0/0/9	0/0/9	0/0/9	0/0/9	0/0/9	0/0/9	0/0/9	0/0/9	**9/0/0**
	**50**	**W|T|L**	0/0/9	0/0/9	0/0/9	0/0/9	0/0/9	0/0/9	2/0/7	0/0/9	0/0/9	0/0/9	**7/0/2**
	**100**	**W|T|L**	0/0/9	0/0/9	0/0/9	0/0/9	0/0/9	0/0/9	1/0/8	0/0/9	0/0/9	0/0/9	**8/0/1**

**Table 3:** Comparison of optimization results obtained from hybrid test functions.

F	D	Metrics	SA (1983)	CGA (2000)	GWO (2014)	MFO (2015)	WOA (2016)	LMFO (2016)	WCMFO (2019)	ChOA (2020)	AOA (2021)	SMFO (2021)	I-MFO
F_11_	30	Avg	3.124 × 10^3^	5.262 × 10^3^	1.406 × 10^3^	3.749 × 10^3^	1.462 × 10^3^	1.292 × 10^3^	1.336 × 10^3^	3.361 × 10^3^	3.325 × 10^3^	5.265 × 10^3^	**1.188 × 10^3^**
		Min	2.512 × 10^3^	3.284 × 10^3^	1.271 × 10^3^	1.363 × 10^3^	1.282 × 10^3^	1.177 × 10^3^	1.254 × 10^3^	1.731 × 10^3^	1.739 × 10^3^	2.547 × 10^3^	1.119 × 10^3^
	50	Avg	1.128 × 10^4^	1.978 × 10^4^	3.078 × 10^3^	7.297 × 10^3^	1.591 × 10^3^	1.532 × 10^3^	1.491 × 10^3^	8.609 × 10^3^	1.605 × 10^4^	1.426 × 10^4^	**1.326 × 10^3^**
		Min	9.143 × 10^3^	1.250 × 10^4^	1.480 × 10^3^	1.574 × 10^3^	1.421 × 10^3^	1.380 × 10^3^	1.344 × 10^3^	6.220 × 10^3^	9.287 × 10^3^	8.985 × 10^3^	1.212 × 10^3^
	100	Avg	1.883 × 10^5^	2.174 × 10^5^	3.531 × 10^4^	1.257 × 10^5^	7.762 × 10^3^	3.319 × 10^3^	2.191 × 10^3^	7.211 × 10^4^	1.625 × 10^5^	2.086 × 10^5^	**1.776 × 10^3^**
		Min	1.465 × 10^5^	1.629 × 10^5^	1.647 × 10^4^	2.137 × 10^4^	4.463 × 10^3^	2.955 × 10^3^	1.840 × 10^3^	6.100 × 10^4^	1.167 × 10^5^	1.399 × 10^5^	1.464 × 10^3^
F_12_	30	Avg	7.895 × 10^8^	3.540 × 10^9^	3.900 × 10^7^	6.158 × 10^7^	3.770 × 10^7^	5.460 × 10^6^	1.254 × 10^6^	3.360 × 10^9^	7.828 × 10^9^	4.462 × 10^9^	**2.499 × 10^5^**
		Min	3.859 × 10^8^	1.934 × 10^9^	2.109 × 10^6^	7.305 × 10^4^	2.509 × 10^6^	1.046 × 10^6^	3.718 × 10^4^	6.620 × 10^8^	3.034 × 10^9^	1.749 × 10^9^	5.318 × 10^4^
	50	Avg	9.643 × 10^9^	2.939 × 10^10^	4.764 × 10^8^	2.475 × 10^9^	1.861 × 10^8^	4.882 × 10^7^	7.229 × 10^6^	1.887 × 10^10^	5.350 × 10^10^	3.075 × 10^10^	**1.599 × 10^6^**
		Min	5.659 × 10^9^	2.161 × 10^10^	7.558 × 10^7^	1.646 × 10^7^	5.114 × 10^7^	2.169 × 10^7^	1.549 × 10^6^	1.045 × 10^10^	2.948 × 10^10^	1.600 × 10^10^	3.874 × 10^5^
	100	Avg	6.862 × 10^10^	1.398 × 10^11^	4.919 × 10^9^	3.523 × 10^10^	6.875 × 10^8^	3.370 × 10^8^	3.428 × 10^7^	6.764 × 10^10^	1.766 × 10^11^	9.544 × 10^10^	**3.131 × 10^6^**
		Min	5.696 × 10^10^	1.132 × 10^11^	1.450 × 10^9^	1.435 × 10^10^	2.918 × 10^8^	2.296 × 10^8^	3.806 × 10^6^	4.928 × 10^10^	1.296 × 10^11^	5.096 × 10^10^	1.250 × 10^6^
F_13_	30	Avg	9.518 × 10^7^	8.620 × 10^8^	8.368 × 10^5^	7.958 × 10^6^	1.463 × 10^5^	4.494 × 10^5^	1.047 × 10^5^	8.863 × 10^8^	4.348 × 10^4^	8.738 × 10^8^	**1.994 × 10^4^**
		Min	4.048 × 10^7^	3.361 × 10^8^	1.991 × 10^4^	1.122 × 10^4^	2.283 × 10^4^	2.705 × 10^5^	1.436 × 10^4^	3.327 × 10^7^	2.158 × 10^4^	2.189 × 10^8^	1.396 × 10^3^
	50	Avg	1.722 × 10^9^	8.709 × 10^9^	1.532 × 10^8^	2.427 × 10^8^	1.657 × 10^5^	2.510 × 10^6^	8.895 × 10^4^	5.260 × 10^9^	3.917 × 10^9^	1.288 × 10^10^	**1.434 × 10^4^**
		Min	1.103 × 10^9^	5.824 × 10^9^	1.312 × 10^5^	1.136 × 10^5^	4.764 × 10^4^	1.415 × 10^6^	2.174 × 10^4^	5.002 × 10^8^	1.041 × 10^7^	1.435 × 10^9^	1.582 × 10^3^
	100	Avg	8.436 × 10^9^	2.634 × 10^10^	4.163 × 10^8^	4.053 × 10^9^	8.423 × 10^4^	1.168 × 10^7^	1.378 × 10^5^	1.915 × 10^10^	3.479 × 10^10^	1.965 × 10^10^	**1.105 × 10^4^**
		Min	6.140 × 10^9^	1.992 × 10^10^	1.579 × 10^6^	2.629 × 10^8^	3.701 × 10^4^	9.896 × 10^6^	3.658 × 10^4^	1.137 × 10^10^	2.155 × 10^10^	1.073 × 10^10^	1.651 × 10^3^
F_14_	30	Avg	1.022 × 10^5^	3.619 × 10^5^	1.438 × 10^5^	8.969 × 10^4^	9.075 × 10^5^	2.614 × 10^4^	2.073 × 10^4^	3.244 × 10^5^	4.223 × 10^4^	1.548 × 10^6^	**1.671 × 10^4^**
		Min	3.932 × 10^4^	7.165 × 10^4^	3.679 × 10^3^	2.197 × 10^3^	1.364 × 10^5^	2.724 × 10^3^	6.252 × 10^3^	4.503 × 10^4^	2.213 × 10^3^	7.879 × 10^4^	5.615 × 10^3^
	50	Avg	1.285 × 10^6^	5.160 × 10^6^	4.016 × 10^5^	3.086 × 10^5^	6.358 × 10^5^	1.086 × 10^5^	**8.151 × 10^4^**	1.206 × 10^6^	3.933 × 10^5^	2.634 × 10^7^	8.426 × 10^4^
		Min	8.972 × 10^5^	2.700 × 10^6^	4.749 × 10^4^	1.071 × 10^4^	9.639 × 10^4^	2.360 × 10^4^	1.194 × 10^4^	5.706 × 10^5^	4.727 × 10^4^	8.185 × 10^5^	8.798 × 10^3^
	100	Avg	2.710 × 10^7^	6.024 × 10^7^	3.480 × 10^6^	7.558 × 10^6^	1.876 × 10^6^	1.207 × 10^6^	3.627 × 10^5^	7.928 × 10^6^	2.267 × 10^7^	3.200 × 10^7^	**3.439 × 10^5^**
		Min	2.008 × 10^7^	3.524 × 10^7^	1.056 × 10^6^	3.097 × 10^5^	6.461 × 10^5^	1.938 × 10^5^	1.387 × 10^5^	4.302 × 10^6^	4.241 × 10^6^	7.455 × 10^6^	1.557 × 10^5^
F_15_	30	Avg	2.966 × 10^6^	4.746 × 10^7^	3.637 × 10^5^	3.412 × 10^4^	8.683 × 10^4^	9.006 × 10^4^	3.448 × 10^4^	5.434 × 10^6^	2.498 × 10^4^	4.469 × 10^7^	**1.983 × 10^4^**
		Min	1.113 × 10^6^	6.971 × 10^6^	1.847 × 10^4^	3.640 × 10^3^	1.368 × 10^4^	5.002 × 10^4^	2.547 × 10^3^	1.019 × 10^6^	1.454 × 10^4^	2.375 × 10^6^	2.006 × 10^3^
	50	Avg	1.739 × 10^8^	1.276 × 10^9^	9.314 × 10^6^	2.145 × 10^7^	7.839 × 10^4^	5.206 × 10^5^	7.164 × 10^4^	1.070 × 10^8^	3.131 × 10^4^	8.129 × 10^8^	**9.247 × 10^3^**
		Min	6.074 × 10^7^	5.105 × 10^8^	1.565 × 10^4^	4.235 × 10^4^	2.225 × 10^4^	3.594 × 10^5^	1.422 × 10^4^	5.379 × 10^7^	1.979 × 10^4^	1.237 × 10^8^	1.622 × 10^3^
	100	Avg	2.250 × 10^9^	8.426 × 10^9^	9.478 × 10^7^	1.045 × 10^9^	2.527 × 10^5^	2.824 × 10^6^	9.337 × 10^4^	4.851 × 10^9^	4.659 × 10^9^	8.332 × 10^9^	**7.383 × 10^3^**
		Min	1.743 × 10^9^	6.068 × 10^9^	5.864 × 10^5^	1.058 × 10^5^	2.549 × 10^4^	1.949 × 10^6^	1.223 × 10^4^	1.096 × 10^9^	1.070 × 10^9^	1.272 × 10^9^	1.752 × 10^3^
F_16_	30	Avg	3.496 × 10^3^	4.179 × 10^3^	2.287 × 10^3^	2.995 × 10^3^	3.519 × 10^3^	2.640 × 10^3^	2.807 × 10^3^	3.475 × 10^3^	3.676 × 10^3^	4.402 × 10^3^	**1.928 × 10^3^**
		Min	3.252 × 10^3^	3.709 × 10^3^	1.744 × 10^3^	2.487 × 10^3^	2.728 × 10^3^	2.110 × 10^3^	2.095 × 10^3^	2.940 × 10^3^	2.867 × 10^3^	3.607 × 10^3^	1.617 × 10^3^
	50	Avg	5.575 × 10^3^	6.744 × 10^3^	2.791 × 10^3^	4.150 × 10^3^	4.689 × 10^3^	3.621 × 10^3^	3.778 × 10^3^	5.240 × 10^3^	6.261 × 10^3^	6.930 × 10^3^	**2.546 × 10^3^**
		Min	5.216 × 10^3^	6.027 × 10^3^	2.209 × 10^3^	3.133 × 10^3^	3.895 × 10^3^	2.949 × 10^3^	3.014 × 10^3^	4.488 × 10^3^	3.693 × 10^3^	5.302 × 10^3^	2.186 × 10^3^
	100	Avg	1.236 × 10^4^	1.660 × 10^4^	5.610 × 10^3^	8.085 × 10^3^	9.811 × 10^3^	6.439 × 10^3^	6.869 × 10^3^	1.231 × 10^4^	1.814 × 10^4^	1.679 × 10^4^	**4.533 × 10^3^**
		Min	1.111 × 10^4^	1.563 × 10^4^	4.748 × 10^3^	6.389 × 10^3^	7.512 × 10^3^	5.301 × 10^3^	4.978 × 10^3^	1.047 × 10^4^	1.301 × 10^4^	1.394 × 10^4^	3.471 × 10^3^
F_17_	30	Avg	2.410 × 10^3^	2.789 × 10^3^	1.956 × 10^3^	2.411 × 10^3^	2.520 × 10^3^	2.203 × 10^3^	2.315 × 10^3^	2.598 × 10^3^	2.620 × 10^3^	2.752 × 10^3^	**1.875 × 10^3^**
		Min	2.242 × 10^3^	2.467 × 10^3^	1.777 × 10^3^	1.975 × 10^3^	1.931 × 10^3^	1.801 × 10^3^	1.942 × 10^3^	2.275 × 10^3^	2.085 × 10^3^	2.359 × 10^3^	1.736 × 10^3^
	50	Avg	4.770 × 10^3^	5.784 × 10^3^	2.676 × 10^3^	3.708 × 10^3^	3.892 × 10^3^	3.155 × 10^3^	3.758 × 10^3^	4.205 × 10^3^	4.226 × 10^3^	5.316 × 10^3^	**2.573 × 10^3^**
		Min	4.087 × 10^3^	4.805 × 10^3^	2.257 × 10^3^	2.866 × 10^3^	3.106 × 10^3^	2.538 × 10^3^	2.931 × 10^3^	3.304 × 10^3^	3.228 × 10^3^	3.873 × 10^3^	2.176 × 10^3^
	100	Avg	1.132 × 10^4^	9.223 × 10^4^	4.439 × 10^3^	7.668 × 10^3^	7.212 × 10^3^	5.693 × 10^3^	6.345 × 10^3^	1.240 × 10^4^	2.886 × 10^5^	4.082 × 10^5^	**4.247 × 10^3^**
		Min	1.036 × 10^4^	1.996 × 10^4^	3.338 × 10^3^	5.623 × 10^3^	5.421 × 10^3^	4.630 × 10^3^	4.935 × 10^3^	9.483 × 10^3^	1.665 × 10^4^	1.263 × 10^4^	2.980 × 10^3^
F_18_	30	Avg	2.207 × 10^6^	7.273 × 10^6^	6.631 × 10^5^	3.177 × 10^6^	2.408 × 10^6^	3.682 × 10^5^	1.734 × 10^5^	1.487 × 10^6^	7.850 × 10^5^	2.844 × 10^7^	**8.793 × 10^4^**
		Min	1.112 × 10^6^	1.967 × 10^6^	8.000 × 10^4^	3.737 × 10^4^	1.933 × 10^5^	8.629 × 10^4^	3.793 × 10^4^	4.340 × 10^5^	1.205 × 10^5^	2.007 × 10^6^	3.279 × 10^3^
	50	Avg	1.242 × 10^7^	4.494 × 10^7^	3.300 × 10^6^	3.443 × 10^6^	4.272 × 10^6^	7.009 × 10^5^	4.064 × 10^5^	8.349 × 10^6^	2.081 × 10^7^	7.061 × 10^7^	**3.192 × 10^5^**
		Min	5.637 × 10^6^	1.275 × 10^7^	2.968 × 10^5^	1.807 × 10^5^	1.009 × 10^6^	3.224 × 10^5^	1.508 × 10^5^	3.517 × 10^6^	8.364 × 10^5^	1.412 × 10^7^	3.532 × 10^4^
	100	Avg	5.093 × 10^7^	1.121 × 10^8^	4.158 × 10^6^	1.162 × 10^7^	2.020 × 10^6^	2.306 × 10^6^	**8.326 × 10^5^**	1.088 × 10^7^	3.135 × 10^7^	5.663 × 10^7^	1.164 × 10^6^
		Min	3.392 × 10^7^	6.823 × 10^7^	7.431 × 10^5^	4.881 × 10^5^	8.476 × 10^5^	1.032 × 10^6^	3.782 × 10^5^	5.042 × 10^6^	9.728 × 10^6^	9.819 × 10^6^	2.003 × 10^5^
F_19_	30	Avg	1.278 × 10^7^	9.064 × 10^7^	2.913 × 10^5^	4.071 × 10^6^	2.647 × 10^6^	6.193 × 10^4^	3.223 × 10^4^	4.950 × 10^7^	1.071 × 10^6^	1.072 × 10^8^	**2.012 × 10^4^**
		Min	6.005 × 10^6^	3.289 × 10^7^	9.466 × 10^3^	2.093 × 10^3^	1.744 × 10^5^	1.764 × 10^4^	2.168 × 10^3^	2.507 × 10^6^	8.696 × 10^5^	5.192 × 10^6^	1.940 × 10^3^
	50	Avg	9.348 × 10^7^	6.178 × 10^8^	2.362 × 10^6^	6.151 × 10^6^	2.457 × 10^6^	2.445 × 10^5^	2.361 × 10^4^	3.031 × 10^8^	4.614 × 10^5^	9.214 × 10^8^	**1.409 × 10^4^**
		Min	4.313 × 10^7^	2.829 × 10^8^	6.908 × 10^4^	5.030 × 10^3^	1.534 × 10^5^	1.272 × 10^5^	2.700 × 10^3^	3.918 × 10^7^	4.438 × 10^5^	1.580 × 10^8^	2.081 × 10^3^
	100	Avg	2.062 × 10^9^	8.505 × 10^9^	1.003 × 10^8^	3.561 × 10^8^	1.528 × 10^7^	4.435 × 10^6^	7.032 × 10^4^	3.211 × 10^9^	4.723 × 10^9^	5.975 × 10^9^	**1.009 × 10^4^**
		Min	1.411 × 10^9^	6.413 × 10^9^	2.250 × 10^6^	2.761 × 10^6^	5.273 × 10^6^	2.123 × 10^6^	1.223 × 10^4^	7.255 × 10^8^	1.529 × 10^9^	2.984 × 10^9^	2.081 × 10^3^
F_20_	30	Avg	2.533 × 10^3^	2.656 × 10^3^	2.288 × 10^3^	2.600 × 10^3^	2.702 × 10^3^	2.498 × 10^3^	2.468 × 10^3^	2.921 × 10^3^	2.638 × 10^3^	2.847 × 10^3^	**2.116 × 10^3^**
		Min	2.461 × 10^3^	2.473 × 10^3^	2.154 × 10^3^	2.215 × 10^3^	2.327 × 10^3^	2.180 × 10^3^	2.072 × 10^3^	2.560 × 10^3^	2.327 × 10^3^	2.454 × 10^3^	2.018 × 10^3^
	50	Avg	3.891 × 10^3^	3.930 × 10^3^	2.736 × 10^3^	3.557 × 10^3^	3.628 × 10^3^	3.060 × 10^3^	3.431 × 10^3^	3.932 × 10^3^	3.346 × 10^3^	3.929 × 10^3^	**2.297 × 10^3^**
		Min	3.535 × 10^3^	3.587 × 10^3^	2.422 × 10^3^	2.897 × 10^3^	2.664 × 10^3^	2.586 × 10^3^	2.655 × 10^3^	3.576 × 10^3^	2.634 × 10^3^	3.493 × 10^3^	2.097 × 10^3^
	100	Avg	7.466 × 10^3^	7.382 × 10^3^	4.469 × 10^3^	5.692 × 10^3^	5.875 × 10^3^	5.054 × 10^3^	5.740 × 10^3^	6.931 × 10^3^	5.751 × 10^3^	6.923 × 10^3^	**3.566 × 10^3^**
		Min	6.910 × 10^3^	6.809 × 10^3^	3.301 × 10^3^	4.194 × 10^3^	4.326 × 10^3^	4.139 × 10^3^	4.438 × 10^3^	6.030 × 10^3^	4.700 × 10^3^	6.187 × 10^3^	3.093 × 10^3^
**Ranking**	**30**	**W|T|L**	0/0/10	0/0/10	0/0/10	0/0/10	0/0/10	0/0/10	0/0/10	0/0/10	0/0/10	0/0/10	**10/0/0**
	**50**	**W|T|L**	0/0/10	0/0/10	0/0/10	0/0/10	0/0/10	0/0/10	1/0/9	0/0/10	0/0/10	0/0/10	**9/0/1**
	**100**	**W|T|L**	0/0/10	0/0/10	0/0/10	0/0/10	0/0/10	0/0/10	1/0/9	0/0/10	0/0/10	0/0/10	**9/0/1**

**Table 4:** Comparison of optimization results obtained from composition test functions.

F	D	Metrics	SA (1983)	CGA (2000)	GWO (2014)	MFO (2015)	WOA (2016)	LMFO (2016)	WCMFO (2019)	ChOA (2020)	AOA (2021)	SMFO (2021)	I-MFO
F_21_	30	Avg	2.593 × 10^3^	2.655 × 10^3^	2.383 × 10^3^	2.476 × 10^3^	2.558 × 10^3^	2.439 × 10^3^	2.493 × 10^3^	2.565 × 10^3^	2.604 × 10^3^	2.653 × 10^3^	**2.363 × 10^3^**
		Min	2.565 × 10^3^	2.626 × 10^3^	2.352 × 10^3^	2.421 × 10^3^	2.463 × 10^3^	2.378 × 10^3^	2.398 × 10^3^	2.503 × 10^3^	2.515 × 10^3^	2.551 × 10^3^	2.334 × 10^3^
	50	Avg	2.945 × 10^3^	3.065 × 10^3^	2.485 × 10^3^	2.694 × 10^3^	2.888 × 10^3^	2.609 × 10^3^	2.694 × 10^3^	2.886 × 10^3^	3.002 × 10^3^	3.064 × 10^3^	**2.430 × 10^3^**
		Min	2.893 × 10^3^	3.026 × 10^3^	2.440 × 10^3^	2.575 × 10^3^	2.744 × 10^3^	2.542 × 10^3^	2.580 × 10^3^	2.819 × 10^3^	2.885 × 10^3^	2.935 × 10^3^	2.399 × 10^3^
	100	Avg	4.058 × 10^3^	4.393 × 10^3^	2.845 × 10^3^	3.594 × 10^3^	3.884 × 10^3^	3.280 × 10^3^	3.539 × 10^3^	4.044 × 10^3^	4.581 × 10^3^	4.394 × 10^3^	**2.738 × 10^3^**
		Min	3.956 × 10^3^	4.275 × 10^3^	2.751 × 10^3^	3.262 × 10^3^	3.502 × 10^3^	3.106 × 10^3^	3.233 × 10^3^	3.804 × 10^3^	4.161 × 10^3^	4.128 × 10^3^	2.652 × 10^3^
F_22_	30	Avg	6.618 × 10^3^	6.787 × 10^3^	4.413 × 10^3^	5.842 × 10^3^	5.949 × 10^3^	5.006 × 10^3^	6.637 × 10^3^	9.124 × 10^3^	7.785 × 10^3^	8.654 × 10^3^	**2.889 × 10^3^**
		Min	4.837 × 10^3^	5.363 × 10^3^	2.420 × 10^3^	3.150 × 10^3^	2.315 × 10^3^	2.325 × 10^3^	5.330 × 10^3^	8.503 × 10^3^	5.492 × 10^3^	5.677 × 10^3^	2.300 × 10^3^
	50	Avg	1.569 × 10^4^	1.600 × 10^4^	8.634 × 10^3^	1.029 × 10^4^	1.208 × 10^4^	8.858 × 10^3^	1.001 × 10^4^	1.655 × 10^4^	1.468 × 10^4^	1.616 × 10^4^	**6.525 × 10^3^**
		Min	1.464 × 10^4^	1.489 × 10^4^	7.065 × 10^3^	7.958 × 10^3^	8.721 × 10^3^	7.176 × 10^3^	8.609 × 10^3^	1.554 × 10^4^	1.304 × 10^4^	1.529 × 10^4^	5.551 × 10^3^
	100	Avg	3.336 × 10^4^	3.346 × 10^4^	1.777 × 10^4^	2.032 × 10^4^	2.397 × 10^4^	1.948 × 10^4^	1.943 × 10^4^	3.374 × 10^4^	3.092 × 10^4^	3.277 × 10^4^	**1.393 × 10^4^**
		Min	3.264 × 10^4^	3.169 × 10^4^	1.413 × 10^4^	1.778 × 10^4^	2.087 × 10^4^	1.791 × 10^4^	1.671 × 10^4^	3.233 × 10^4^	2.790 × 10^4^	3.043 × 10^4^	1.189 × 10^4^
F_23_	30	Avg	2.916 × 10^3^	3.149 × 10^3^	2.731 × 10^3^	2.801 × 10^3^	3.032 × 10^3^	2.759 × 10^3^	2.785 × 10^3^	3.011 × 10^3^	3.312 × 10^3^	3.283 × 10^3^	**2.700 × 10^3^**
		Min	2.819 × 10^3^	3.102 × 10^3^	2.695 × 10^3^	2.762 × 10^3^	2.886 × 10^3^	2.710 × 10^3^	2.721 × 10^3^	2.930 × 10^3^	3.093 × 10^3^	3.027 × 10^3^	2.680 × 10^3^
	50	Avg	3.380 × 10^3^	3.816 × 10^3^	2.907 × 10^3^	3.135 × 10^3^	3.592 × 10^3^	3.027 × 10^3^	3.104 × 10^3^	3.515 × 10^3^	4.310 × 10^3^	3.929 × 10^3^	**2.858 × 10^3^**
		Min	3.345 × 10^3^	3.644 × 10^3^	2.835 × 10^3^	3.046 × 10^3^	3.377 × 10^3^	2.990 × 10^3^	2.980 × 10^3^	3.373 × 10^3^	3.850 × 10^3^	3.594 × 10^3^	2.820 × 10^3^
	100	Avg	4.322 × 10^3^	5.475 × 10^3^	3.405 × 10^3^	3.716 × 10^3^	4.823 × 10^3^	3.475 × 10^3^	3.545 × 10^3^	4.661 × 10^3^	6.745 × 10^3^	6.024 × 10^3^	**3.035 × 10^3^**
		Min	4.238 × 10^3^	5.225 × 10^3^	3.289 × 10^3^	3.547 × 10^3^	4.263 × 10^3^	3.366 × 10^3^	3.306 × 10^3^	4.424 × 10^3^	6.011 × 10^3^	5.104 × 10^3^	2.971 × 10^3^
F_24_	30	Avg	3.084 × 10^3^	3.342 × 10^3^	2.904 × 10^3^	2.974 × 10^3^	3.167 × 10^3^	2.927 × 10^3^	2.978 × 10^3^	3.201 × 10^3^	3.682 × 10^3^	3.433 × 10^3^	**2.871 × 10^3^**
		Min	3.063 × 10^3^	3.270 × 10^3^	2.855 × 10^3^	2.910 × 10^3^	3.021 × 10^3^	2.897 × 10^3^	2.928 × 10^3^	3.128 × 10^3^	3.473 × 10^3^	3.217 × 10^3^	2.852 × 10^3^
	50	Avg	3.459 × 10^3^	4.039 × 10^3^	3.087 × 10^3^	3.227 × 10^3^	3.733 × 10^3^	3.136 × 10^3^	3.231 × 10^3^	3.713 × 10^3^	4.749 × 10^3^	4.359 × 10^3^	**3.008 × 10^3^**
		Min	3.416 × 10^3^	3.856 × 10^3^	3.000 × 10^3^	3.152 × 10^3^	3.545 × 10^3^	3.071 × 10^3^	3.135 × 10^3^	3.588 × 10^3^	4.340 × 10^3^	3.875 × 10^3^	2.964 × 10^3^
	100	Avg	5.059 × 10^3^	8.073 × 10^3^	3.962 × 10^3^	4.272 × 10^3^	5.854 × 10^3^	4.086 × 10^3^	4.293 × 10^3^	5.913 × 10^3^	1.065 × 10^4^	8.875 × 10^3^	**3.651 × 10^3^**
		Min	4.961 × 10^3^	7.435 × 10^3^	3.819 × 10^3^	4.124 × 10^3^	5.238 × 10^3^	3.976 × 10^3^	4.048 × 10^3^	5.524 × 10^3^	8.928 × 10^3^	6.869 × 10^3^	3.556 × 10^3^
F_25_	30	Avg	4.148 × 10^3^	5.436 × 10^3^	2.957 × 10^3^	3.107 × 10^3^	2.945 × 10^3^	2.889 × 10^3^	**2.887 × 10^3^**	4.099 × 10^3^	4.426 × 10^3^	3.940 × 10^3^	2.888 × 10^3^
		Min	3.828 × 10^3^	4.615 × 10^3^	2.913 × 10^3^	2.889 × 10^3^	2.898 × 10^3^	2.888 × 10^3^	2.884 × 10^3^	3.456 × 10^3^	3.635 × 10^3^	3.463 × 10^3^	2.887 × 10^3^
	50	Avg	1.054 × 10^4^	1.824 × 10^4^	3.371 × 10^3^	4.930 × 10^3^	3.155 × 10^3^	3.043 × 10^3^	3.041 × 10^3^	8.621 × 10^3^	1.388 × 10^4^	1.083 × 10^4^	**3.000 × 10^3^**
		Min	7.933 × 10^3^	1.397 × 10^4^	3.055 × 10^3^	3.159 × 10^3^	3.039 × 10^3^	2.994 × 10^3^	2.962 × 10^3^	6.928 × 10^3^	1.101 × 10^4^	7.534 × 10^3^	2.978 × 10^3^
	100	Avg	5.160 × 10^4^	5.583 × 10^4^	5.277 × 10^3^	1.123 × 10^4^	3.590 × 10^3^	3.456 × 10^3^	3.321 × 10^3^	1.363 × 10^4^	2.328 × 10^4^	2.002 × 10^4^	**3.262 × 10^3^**
		Min	4.633 × 10^4^	4.701 × 10^4^	4.686 × 10^3^	4.792 × 10^3^	3.464 × 10^3^	3.365 × 10^3^	3.206 × 10^3^	1.142 × 10^4^	1.986 × 10^4^	1.680 × 10^4^	3.116 × 10^3^
F_26_	30	Avg	6.408 × 10^3^	8.876 × 10^3^	4.424 × 10^3^	5.689 × 10^3^	7.599 × 10^3^	5.012 × 10^3^	5.447 × 10^3^	6.328 × 10^3^	9.412 × 10^3^	8.871 × 10^3^	**4.300 × 10^3^**
		Min	5.542 × 10^3^	7.735 × 10^3^	3.954 × 10^3^	4.921 × 10^3^	5.975 × 10^3^	4.607 × 10^3^	4.955 × 10^3^	5.882 × 10^3^	7.702 × 10^3^	5.057 × 10^3^	2.900 × 10^3^
	50	Avg	1.063 × 10^4^	1.594 × 10^4^	5.735 × 10^3^	8.121 × 10^3^	1.306 × 10^4^	7.041 × 10^3^	8.059 × 10^3^	1.028 × 10^4^	1.546 × 10^4^	1.587 × 10^4^	**5.179 × 10^3^**
		Min	1.002 × 10^4^	1.454 × 10^4^	5.192 × 10^3^	6.910 × 10^3^	9.977 × 10^3^	6.161 × 10^3^	7.062 × 10^3^	9.047 × 10^3^	1.326 × 10^4^	1.396 × 10^4^	4.512 × 10^3^
	100	Avg	2.452 × 10^4^	4.461 × 10^4^	1.263 × 10^4^	1.741 × 10^4^	3.111 × 10^4^	1.493 × 10^4^	1.752 × 10^4^	2.492 × 10^4^	4.995 × 10^4^	4.315 × 10^4^	**9.748 × 10^3^**
		Min	2.363 × 10^4^	4.099 × 10^4^	1.124 × 10^4^	1.526 × 10^4^	2.326 × 10^4^	1.333 × 10^4^	1.518 × 10^4^	2.276 × 10^4^	4.219 × 10^4^	3.583 × 10^4^	9.123 × 10^3^
F_27_	30	Avg	3.279 × 10^3^	3.667 × 10^3^	3.229 × 10^3^	3.236 × 10^3^	3.346 × 10^3^	3.221 × 10^3^	3.228 × 10^3^	3.493 × 10^3^	4.286 × 10^3^	3.688 × 10^3^	**3.213 × 10^3^**
		Min	3.250 × 10^3^	3.497 × 10^3^	3.212 × 10^3^	3.208 × 10^3^	3.282 × 10^3^	3.200 × 10^3^	3.201 × 10^3^	3.355 × 10^3^	3.633 × 10^3^	3.397 × 10^3^	3.184 × 10^3^
	50	Avg	3.730 × 10^3^	5.183 × 10^3^	3.471 × 10^3^	3.550 × 10^3^	4.305 × 10^3^	3.356 × 10^3^	3.504 × 10^3^	4.272 × 10^3^	6.565 × 10^3^	5.306 × 10^3^	**3.337 × 10^3^**
		Min	3.669 × 10^3^	4.697 × 10^3^	3.342 × 10^3^	3.407 × 10^3^	3.678 × 10^3^	3.249 × 10^3^	3.377 × 10^3^	3.997 × 10^3^	5.687 × 10^3^	4.453 × 10^3^	3.231 × 10^3^
	100	Avg	4.858 × 10^3^	8.855 × 10^3^	3.854 × 10^3^	3.867 × 10^3^	4.945 × 10^3^	3.500 × 10^3^	3.607 × 10^3^	5.656 × 10^3^	1.177 × 10^4^	9.335 × 10^3^	**3.467 × 10^3^**
		Min	4.700 × 10^3^	7.573 × 10^3^	3.594 × 10^3^	3.655 × 10^3^	3.909 × 10^3^	3.389 × 10^3^	3.482 × 10^3^	5.033 × 10^3^	9.541 × 10^3^	5.884 × 10^3^	3.381 × 10^3^
F_28_	30	Avg	4.040 × 10^3^	5.557 × 10^3^	3.339 × 10^3^	3.721 × 10^3^	3.303 × 10^3^	3.255 × 10^3^	**3.194 × 10^3^**	4.295 × 10^3^	5.958 × 10^3^	5.524 × 10^3^	3.226 × 10^3^
		Min	3.927 × 10^3^	4.829 × 10^3^	3.269 × 10^3^	3.318 × 10^3^	3.269 × 10^3^	3.209 × 10^3^	3.100 × 10^3^	3.565 × 10^3^	4.603 × 10^3^	4.419 × 10^3^	3.155 × 10^3^
	50	Avg	8.098 × 10^3^	1.147 × 10^4^	3.873 × 10^3^	8.080 × 10^3^	3.424 × 10^3^	3.316 × 10^3^	3.298 × 10^3^	6.101 × 10^3^	1.102 × 10^4^	9.557 × 10^3^	**3.278 × 10^3^**
		Min	6.921 × 10^3^	8.816 × 10^3^	3.653 × 10^3^	5.324 × 10^3^	3.344 × 10^3^	3.274 × 10^3^	3.259 × 10^3^	5.216 × 10^3^	9.574 × 10^3^	8.008 × 10^3^	3.259 × 10^3^
	100	Avg	2.499 × 10^4^	3.958 × 10^4^	6.692 × 10^3^	1.749 × 10^4^	3.721 × 10^3^	1.149 × 10^4^	7.644 × 10^3^	1.204 × 10^4^	2.938 × 10^4^	2.320 × 10^4^	**3.357 × 10^3^**
		Min	2.393 × 10^4^	3.584 × 10^4^	4.771 × 10^3^	1.485 × 10^4^	3.598 × 10^3^	3.439 × 10^3^	3.333 × 10^3^	9.983 × 10^3^	2.587 × 10^4^	1.831 × 10^4^	3.321 × 10^3^
F_29_	30	Avg	4.387 × 10^3^	5.321 × 10^3^	3.645 × 10^3^	4.003 × 10^3^	4.751 × 10^3^	3.785 × 10^3^	3.965 × 10^3^	4.348 × 10^3^	5.689 × 10^3^	5.698 × 10^3^	**3.465 × 10^3^**
		Min	4.114 × 10^3^	4.426 × 10^3^	3.459 × 10^3^	3.603 × 10^3^	4.062 × 10^3^	3.596 × 10^3^	3.650 × 10^3^	4.057 × 10^3^	4.626 × 10^3^	4.728 × 10^3^	3.343 × 10^3^
	50	Avg	6.503 × 10^3^	9.644 × 10^3^	4.214 × 10^3^	5.076 × 10^3^	7.281 × 10^3^	4.337 × 10^3^	4.671 × 10^3^	6.906 × 10^3^	1.548 × 10^4^	1.622 × 10^4^	**3.589 × 10^3^**
		Min	5.954 × 10^3^	7.327 × 10^3^	3.750 × 10^3^	4.271 × 10^3^	6.025 × 10^3^	3.820 × 10^3^	3.992 × 10^3^	5.462 × 10^3^	8.398 × 10^3^	8.290 × 10^3^	3.288 × 10^3^
	100	Avg	1.758 × 10^4^	5.024 × 10^4^	7.229 × 10^3^	1.370 × 10^4^	1.413 × 10^4^	6.953 × 10^3^	7.986 × 10^3^	1.940 × 10^4^	8.567 × 10^4^	5.425 × 10^4^	**5.805 × 10^3^**
		Min	1.557 × 10^4^	2.701 × 10^4^	6.385 × 10^3^	7.555 × 10^3^	1.053 × 10^4^	5.760 × 10^3^	7.019 × 10^3^	1.268 × 10^4^	3.350 × 10^4^	1.727 × 10^4^	5.194 × 10^3^
F_30_	30	Avg	1.398 × 10^7^	1.149 × 10^8^	7.020 × 10^6^	3.271 × 10^5^	6.709 × 10^6^	1.579 × 10^5^	2.811 × 10^4^	3.527 × 10^7^	4.703 × 10^7^	3.278 × 10^8^	**1.064 × 10^4^**
		Min	4.880 × 10^6^	2.608 × 10^7^	8.829 × 10^5^	1.393 × 10^4^	4.463 × 10^5^	4.934 × 10^4^	1.582 × 10^4^	1.030 × 10^7^	1.875 × 10^6^	3.212 × 10^7^	5.336 × 10^3^
	50	Avg	3.257 × 10^8^	1.521 × 10^9^	6.713 × 10^7^	8.852 × 10^7^	8.101 × 10^7^	5.293 × 10^6^	2.475 × 10^6^	5.299 × 10^8^	5.682 × 10^8^	2.207 × 10^9^	**1.323 × 10^6^**
		Min	1.906 × 10^8^	7.735 × 10^8^	3.536 × 10^7^	2.389 × 10^6^	4.041 × 10^7^	3.797 × 10^6^	1.155 × 10^6^	1.890 × 10^8^	1.863 × 10^8^	2.782 × 10^8^	7.972 × 10^5^
	100	Avg	3.841 × 10^9^	1.351 × 10^10^	3.958 × 10^8^	1.283 × 10^9^	1.922 × 10^8^	1.283 × 10^7^	1.932 × 10^6^	1.185 × 10^10^	3.055 × 10^10^	1.581 × 10^10^	**7.578 × 10^3^**
		Min	3.148 × 10^9^	8.053 × 10^9^	5.455 × 10^7^	3.821 × 10^7^	7.264 × 10^7^	7.913 × 10^6^	3.637 × 10^5^	8.263 × 10^9^	1.450 × 10^10^	4.669 × 10^9^	5.286 × 10^3^
	**30**	**W|T|L**	0/0/10	0/0/10	0/0/10	0/0/10	0/0/10	0/0/10	2/0/8	0/0/10	0/0/10	0/0/10	**8/0/2**
**Ranking**	**50**	**W|T|L**	0/0/10	0/0/10	0/0/10	0/0/10	0/0/10	0/0/10	0/0/10	0/0/10	0/0/10	0/0/10	**10/0/0**
	**100**	**W|T|L**	0/0/10	0/0/10	0/0/10	0/0/10	0/0/10	0/0/10	0/0/10	0/0/10	0/0/10	0/0/10	**10/0/0**

**Table 5:** The overall effectiveness of the I-MFO and contender algorithms.

Algorithms	SA (W/T/L)	CGA (W/T/L)	GWO (W/T/L)	MFO (W/T/L)	WOA (W/T/L)	LMFO (W/T/L)	WCMFO (W/T/L)	ChOA (W/T/L)	AOA (W/T/L)	SMFO (W/T/L)	I-MFO (W/T/L)
D = 30	0/0/29	0/0/29	0/0/29	0/0/29	0/0/29	0/0/29	2/0/27	0/0/29	0/0/29	0/0/29	27/0/2
D = 50	0/0/29	0/0/29	0/0/29	0/0/29	0/0/29	0/0/29	3/0/26	0/0/29	0/0/29	0/0/29	26/0/3
D = 100	0/0/29	0/0/29	0/0/29	0/0/29	0/0/29	0/0/29	2/0/27	0/0/29	0/0/29	0/0/29	27/0/2
Total	0/0/87	0/0/87	0/0/87	0/0/87	0/0/87	0/0/87	7/0/80	0/0/87	0/0/87	0/0/87	80/0/7
OE	0%	0%	0%	0%	0%	0%	8%	0%	0%	0%	**92%**

**Table 6:** Friedman test for unimodal and multimodal functions of the CEC 2018.

Functions	Unimodal Functions						Multimodal Functions					
Dimensions	30		50		100		30		50		100	
Algorithms	Avg. Rank	Overall Rank	Avg. Rank	Overall Rank	Avg. Rank	Overall Rank	Avg. Rank	Overall Rank	Avg. Rank	Overall Rank	Avg. Rank	Overall Rank
SA	9.22	10	9.77	10	10.50	11	8.67	9	9.45	10	10.15	10
CGA	9.75	11	10.42	11	10.25	10	10.49	11	10.74	11	10.29	11
GWO	4.57	4	5.12	5	4.12	3	2.52	2	2.66	2	2.578	2
MFO	6.30	6	6.07	6	6.30	7	4.65	5	4.67	5	5.26	6
WOA	6.70	7	3.60	4	6.10	5	6.21	6	5.45	6	4.53	4
LMFO	3.45	3	3.57	3	4.15	4	2.99	3	2.85	3	3.29	3
WCMFO	1.77	2	1.62	2	1.70	2	4.61	4	4.52	4	4.67	5
ChOA	6.17	5	6.70	7	6.17	6	7.19	7	7.27	7	7.34	7
AOA	8.12	8	9.02	9	7.80	9	7.67	8	8.04	8	7.98	8
SMFO	8.57	9	8.57	8	7.57	8	9.75	10	9.14	9	8.82	9
I-MFO	**1.35**	**1**	**1.50**	**1**	**1.32**	**1**	**1.20**	**1**	**1.17**	**1**	**1.06**	**1**

**Table 7:** Friedman test for hybrid and composition functions of the CEC 2018.

Functions	Hybrid Functions						Composition Functions					
Dimensions	30		50		100		30		50		100	
Algorithms	Avg. Rank	Overall Rank	Avg. Rank	Overall Rank	Avg. Rank	Overall Rank	Avg. Rank	Overall Rank	Avg. Rank	Overall Rank	Avg. Rank	Overall Rank
SA	7.96	8	8.74	9	8.52	8	7.20	7	8.74	9	7.68	8
CGA	9.94	10	10.19	11	10.20	11	9.50	9	10.19	11	9.65	10
GWO	3.79	2	3.79	3	4.17	5	3.12	2	3.795	3	3.14	2
MFO	3.87	4	4.56	5	5.4	6	4.36	5	4.56	5	4.95	5
WOA	6.49	7	5.14	6	4.04	4	6.05	6	5.14	6	5.83	6
LMFO	4.63	5	4.06	4	3.65	3	3.39	3	4.06	4	3.23	3
WCMFO	3.86	3	3.57	2	2.95	2	3.99	4	3.57	2	3.55	4
ChOA	8.18	9	7.74	8	7.48	7	7.70	8	7.74	8	7.4	7
AOA	5.81	6	6.96	7	8.8	9	9.64	10	6.96	7	10.15	11
SMFO	10.21	11	10.12	10	9.62	10	9.90	11	10.12	10	9.36	9
I-MFO	**1.23**	**1**	**1.10**	**1**	**1.09**	**1**	**1.12**	**1**	**1.10**	**1**	**1.03**	**1**

**Table 8:** Adjusted *p*-values for the Friedman test on different dimensions (I-MFO is the control method).

Dimensions	30		50		100	
Algorithms	Bonferroni *p*-Value	Tukey *p*-Value	Bonferroni *p*-Value	Tukey *p*-Value	Bonferroni *p*-Value	Tukey *p*-Value
SA	7.238 × 10^−8^	7.247 × 10^−8^	7.238 × 10^−8^	7.247 × 10^−8^	7.238 × 10^−8^	7.247 × 10^-08^
CGA	7.238 × 10^−8^	7.247 × 10^−8^	7.238 × 10^−8^	7.247 × 10^−8^	7.238 × 10^−8^	7.247 × 10^−8^
GWO	5.337 × 10^−7^	5.338 × 10^−7^	5.337 × 10^−7^	5.338 × 10^−7^	7.238 × 10^−8^	7.247 × 10^−8^
MFO	5.337 × 10^−7^	5.338 × 10^−7^	7.238 × 10^−8^	7.247 × 10^−8^	7.238 × 10^−8^	7.247 × 10^−8^
WOA	7.238 × 10^−8^	7.247 × 10^−8^	7.238 × 10^−8^	7.247 × 10^−8^	7.238 × 10^−8^	7.247 × 10^−8^
LMFO	3.444 × 10^−6^	3.444 × 10^−6^	5.337 × 10^−7^	5.338 × 10^−7^	5.337 × 10^−7^	5.338 × 10^−7^
WCMFO	1.595 × 10^−3^	1.595 × 10^−3^	3.444 × 10^−6^	3.444 × 10^−6^	3.444 × 10^−6^	3.444 × 10^−6^
ChOA	7.238 × 10^−8^	7.247 × 10^−8^	7.238 × 10^−8^	7.247 × 10^−8^	7.238 × 10^−8^	7.247 × 10^−8^
AOA	5.337 × 10^−7^	5.338 × 10^−7^	7.238 × 10^−8^	7.247 × 10^−8^	7.238 × 10^−8^	7.247 × 10^−8^
SMFO	7.238 × 10^−8^	7.247 × 10^−8^	7.238 × 10^−8^	7.247 × 10^−8^	7.238 × 10^−8^	7.247 × 10^−8^

**Table 9:** Results of the gas transmission compressor design problem.

Algorithms	Optimal Values for Variables				Optimal Cost
	*x*_1_	*x*_2_	*x*_3_	*x*_4_	
SA	46.76	1.62	25.79	0.55	4.390311 × 10^6^
CGA	49.97	20.01	31.47	49.83	1.735023 × 10^7^
GWO	20.00	7.81	20.00	60.00	2.964974 × 10^6^
MFO	50.00	1.18	24.57	0.39	2.964902 × 10^6^
WOA	50.00	1.18	24.86	0.39	2.965002 × 10^6^
LMFO	49.46	1.18	24.64	0.39	2.965456 × 10^6^
WCMFO	50.00	1.18	24.61	0.39	2.964897 × 10^6^
ChOA	50.00	1.19	24.24	0.41	2.966828 × 10^6^
AOA	50.00	1.23	20.00	0.51	3.014615 × 10^6^
SMFO	23.66	1.09	23.66	0.19	3.052254 × 10^6^
I-MFO	50.00	1.18	24.60	0.39	**2.964896 × 10^6^**

**Table 10:** Results of the three-bar truss problem.

Algorithms	Optimal Values for Variables		Optimal Weight
	*x*_1_	*x*_2_	
SA	0.768630	0.474232	2.6482456 × 10^2^
CGA	0.792428	0.397752	2.6390770 × 10^2^
GWO	0.787771	0.410872	2.6389619 × 10^2^
MFO	0.789186	0.406806	2.6389603 × 10^2^
WOA	0.787713	0.410977	2.6389653 × 10^2^
LMFO	0.791713	0.399909	2.6392114 × 10^2^
WCMFO	0.788472	0.408822	2.6389589 × 10^2^
ChOA	0.787802	0.410724	2.6389653 × 10^2^
AOA	0.792789	0.396906	2.6392526 × 10^2^
SMFO	0.792044	0.398859	2.6390973 × 10^2^
I-MFO	0.788792	0.407919	**2.6389585 × 10^2^**

**Table 11:** Results for tension/compression spring design problem.

Algorithms	Optimal Values for Variables			Optimum Weight
	*d*	*D*	*N*	
SA	0.075935	0.993094	3.879891	0.033670
CGA	0.071031	1.019975	1.726076	0.019749
GWO	0.051231	0.345699	11.970135	0.012676
MFO	0.053064	0.390718	9.542437	0.012699
WOA	0.050451	0.327675	13.219341	0.012694
LMFO	0.050000	0.317154	14.107156	0.012771
WCMFO	0.051509	0.352411	11.545969	0.012666
ChOA	0.051069	0.341746	12.251078	0.012702
AOA	0.050000	0.310475	15.000000	0.013195
SMFO	0.050000	0.314692	14.696505	0.013136
I-MFO	0.051710	0.357217	11.259785	**0.012665**

## Data Availability

The data and code used in the research may be obtained from the corresponding author upon request.
